# Neoadjuvant neratinib promotes ferroptosis and inhibits brain metastasis in a novel syngeneic model of spontaneous HER2^+ve^ breast cancer metastasis

**DOI:** 10.1186/s13058-019-1177-1

**Published:** 2019-08-13

**Authors:** Aadya Nagpal, Richard P. Redvers, Xiawei Ling, Scott Ayton, Miriam Fuentes, Elnaz Tavancheh, Irmina Diala, Alshad Lalani, Sherene Loi, Steven David, Robin L. Anderson, Yvonne Smith, Delphine Merino, Delphine Denoyer, Normand Pouliot

**Affiliations:** 1 Matrix Microenvironment & Metastasis Laboratory, Olivia Newton-John Cancer Research Institute, 145 Studley Road, Heidelberg, VIC 3084 Australia; 2 School of Cancer Medicine, La Trobe University, Bundoora, VIC 3086 Australia; 3 Metastasis Research Laboratory, Olivia Newton-John Cancer Research Institute, Heidelberg, VIC 3084 Australia; 4 Metastasis Research Laboratory, Peter MacCallum Cancer Centre, Melbourne, VIC 3000 Australia; 5 Florey Institute of Neuroscience and Mental Health, Parkville, VIC 3052 Australia; 6 Puma Biotechnology, Inc., 10880 Wilshire Blvd, Los Angeles, CA 90024 USA; 7 Translational Breast Cancer Genomics Laboratory, Peter MacCallum Cancer Centre, Melbourne, VIC 3000 Australia; 8 Peter MacCallum Cancer Centre, Moorabbin Campus, East Bentleigh, VIC 3165 Australia; 9 Sir Peter MacCallum Department of Oncology, The University of Melbourne, Melbourne, VIC 3000 Australia; 10 Department of Clinical Pathology, The University of Melbourne, Melbourne, VIC 3000 Australia; 11 Royal College of Surgeons, Dublin, D02 YN77 Ireland; 12 Tumour Progression and Heterogeneity Laboratory, Olivia Newton-John Cancer Research Institute, Heidelberg, VIC 3084 Australia; 13 Molecular Medicine Division, The Walter and ELIZA Hall Institute of Medical Research, Parkville, VIC 3052 Australia; 14 Department of Medical Biology, The University of Melbourne, Melbourne, VIC 3010 Australia

**Keywords:** TBCP-1, HER2-positive breast cancer, Brain metastasis, Syngeneic mouse model, Neratinib, Ferroptosis, Tyrosine kinase inhibitors

## Abstract

**Background:**

Human epidermal growth factor receptor-2 (HER2)-targeted therapies prolong survival in HER2-positive breast cancer patients. Benefit stems primarily from improved control of systemic disease, but up to 50% of patients progress to incurable brain metastases due to acquired resistance and/or limited permeability of inhibitors across the blood-brain barrier. Neratinib, a potent irreversible pan-tyrosine kinase inhibitor, prolongs disease-free survival in the extended adjuvant setting, and several trials evaluating its efficacy alone or combination with other inhibitors in early and advanced HER2-positive breast cancer patients are ongoing. However, its efficacy as a first-line therapy against HER2-positive breast cancer brain metastasis has not been fully explored, in part due to the lack of relevant pre-clinical models that faithfully recapitulate this disease. Here, we describe the development and characterisation of a novel syngeneic model of spontaneous HER2-positive breast cancer brain metastasis (TBCP-1) and its use to evaluate the efficacy and mechanism of action of neratinib.

**Methods:**

TBCP-1 cells were derived from a spontaneous BALB/C mouse mammary tumour and characterised for hormone receptors and HER2 expression by flow cytometry, immunoblotting and immunohistochemistry. Neratinib was evaluated in vitro and in vivo in the metastatic and neoadjuvant setting. Its mechanism of action was examined by transcriptomic profiling, function inhibition assays and immunoblotting.

**Results:**

TBCP-1 cells naturally express high levels of HER2 but lack expression of hormone receptors. TBCP-1 tumours maintain a HER2-positive phenotype in vivo and give rise to a high incidence of spontaneous and experimental metastases in the brain and other organs. Cell proliferation/viability in vitro is inhibited by neratinib and by other HER2 inhibitors, but not by anti-oestrogens, indicating phenotypic and functional similarities to human HER2-positive breast cancer. Mechanistically, neratinib promotes a non-apoptotic form of cell death termed ferroptosis. Importantly, metastasis assays demonstrate that neratinib potently inhibits tumour growth and metastasis, including to the brain, and prolongs survival, particularly when used as a neoadjuvant therapy.

**Conclusions:**

The TBCP-1 model recapitulates the spontaneous spread of HER2-positive breast cancer to the brain seen in patients and provides a unique tool to identify novel therapeutics and biomarkers. Neratinib-induced ferroptosis provides new opportunities for therapeutic intervention. Further evaluation of neratinib neoadjuvant therapy is warranted.

**Electronic supplementary material:**

The online version of this article (10.1186/s13058-019-1177-1) contains supplementary material, which is available to authorized users.

## Background

Breast cancer metastasis to distant organs such as the liver, lung, bone and brain is a leading cause of cancer-related mortality in women and is estimated to account for 626,679 deaths globally in 2018 [1]. Patients with the human epidermal growth factor receptor-2-positive (HER2^+ve^) and the triple-negative (TNBC) subtypes of breast cancer have a higher propensity to develop central nervous system (CNS) metastases [2]. Since no clinically approved biomarkers of brain metastasis are available to prospectively identify high-risk patients, brain lesions are often diagnosed late, when symptomatic and more difficult to treat. Chemotherapy and localised therapies for brain metastases including surgery, whole-brain irradiation and stereotactic radiosurgery, while improving overall median survival, are rarely curative [3]. Hence, more effective therapies are required for incurable brain metastases.

Trastuzumab, a humanised monoclonal antibody targeting HER2, was the first clinically approved targeted therapy for the treatment of HER2^+ve^ breast cancer and is now used routinely as the first-line therapy [4]. Its introduction into the clinic has significantly improved survival outcomes for HER2^+ve^ breast cancer patients. However, not all patients initially respond, and for those who do respond, resistance almost inevitably develops [5]. Moreover, trastuzumab has low permeability across the blood-brain barrier (BBB), and therefore, its benefit is attributed primarily to better control of extra-cranial disease [6–9]. These observations have prompted the development of various strategies to enhance the efficacy of trastuzumab and to overcome resistance, notably with the development of potent small-molecule tyrosine kinase inhibitors (TKIs) currently in the clinic or undergoing clinical trial [8, 10–13].

Neratinib is an irreversible pan-TKI that targets EGFR/HER1, HER2 and HER4. Its potent activity against HER2-overexpressing cells and tumours has been demonstrated clearly in pre-clinical studies, including against trastuzumab or lapatinib-resistant cells and tumours [14–17]. Several trials aimed at identifying the best clinical setting for this inhibitor in early and metastatic breast cancer patients have been completed or are ongoing (reviewed in [18, 19]). Its small size and inhibitory activity on ATP-binding cassette (ABC) transporters have led to the suggestion that neratinib may have a better accumulation in the brain than trastuzumab or other TKIs such as lapatinib [20, 21]. Accordingly, its brain permeability and efficacy against brain metastases are currently under evaluation (NCT01494662). Partial results reported in HER2^+ve^ patients with established brain metastases who experienced progression after one or more lines of CNS local therapy showed a modest CNS objective response rate (8%) to neratinib monotherapy, but almost half of the patients treated with neratinib/capecitabine combination achieved a volumetric reduction of CNS lesions of ≥ 50% [22, 23]. The NEfERT-T phase II trial compared the efficacy of neratinib + paclitaxel versus trastuzumab + paclitaxel in women with locally advanced or metastatic HER2^+ve^ breast cancer [24]. Interestingly, while no difference was found in progression-free survival between the groups, neratinib + paclitaxel combination reduced the incidence of CNS recurrence and delayed the time to brain metastases [24]. In the ExteNET randomised trial (NCT00878709) investigating the efficacy of neratinib after 1 year of trastuzumab adjuvant therapy, neratinib improved disease-free survival but did not reduce the cumulative incidence of CNS recurrence at the 5-year mark [25]. Together, these studies indicate that neratinib may have a place in the clinic for the treatment of patients at risk of brain metastases, but further evaluation in pre-clinical models or in patients is required to determine the most effective treatment regimen to prevent or treat brain metastases.

A major limitation in breast cancer metastasis research is the lack of robust and clinically relevant models of brain metastasis to test novel therapies [26–28]. Most models are human xenografts that lack an intact immune system and require intra-cardiac injection of a bolus of cells for efficient brain metastasis. Given the effect of TKIs on promoting trastuzumab’s antibody-dependent cell-mediated cytotoxicity [10] and the well-documented role of inflammatory cytokines in suppressing tumour immune surveillance and drug response or promoting vessel permeability, immune-competent syngeneic models have greater clinical relevance [29–31]. Syngeneic MMTV-neu and HER2^+ve^ Kunming mouse models circumvent the need for immune-compromised mice but are only weakly metastatic to the lung or liver and not to the brain [32, 33]. Thus, robust syngeneic models that fully recapitulate the spontaneous spread of HER2^+ve^ breast cancer to the brain and other organs would significantly improve our ability to evaluate the efficacy of targeted therapies against HER2^+ve^ brain metastases. Here, we describe a new syngeneic mouse model of HER2^+ve^ breast cancer brain metastasis (TBCP-1) and its response to a panel of TKIs. We demonstrate that the high potency of neratinib is associated with its unique ability to induce cell death by ferroptosis. Furthermore, we present evidence that neoadjuvant neratinib reduces the incidence of brain metastases and provides greater survival benefit than late intervention aimed at treating advanced HER2^+ve^ breast cancer with established brain metastases.

## Methods

### Cell culture and reagents

The 67NR (provided by Dr. F. Miller, Karmanos Cancer Institute, Detroit, MI, USA), the 4T1.2 and the brain-metastatic 4T1Br4 mouse mammary carcinoma cell lines were derived from a BALB/C spontaneous mammary tumour and cultured as described previously [34, 35]. Primary translational breast cancer program-1 (TBCP-1) cells were isolated from a spontaneously arising mammary tumour in a BALB/C mouse (SMT1 kindly donated by Dr. Judy Harmey, Royal College of Surgeons in Ireland) and adapted to culture, from nutrient-rich medium [Dulbecco’s modified Eagle’s medium (DMEM) supplemented with 10% foetal bovine serum (FBS), epidermal growth factor (EGF, 10 ng/ml), insulin (5 μg/ml), glutamine (2 mM), sodium pyruvate (1 mM) and 1% penicillin/streptomycin] to low nutrient medium [DMEM, 10% FBS, sodium pyruvate (1 mM), 1% penicillin/streptomycin] over several months (Additional file 1: Figure S1A). To facilitate ex vivo imaging of metastatic lesions and quantitation of metastatic burden by genomic qPCR, TBCP-1 cells were transduced with a murine stem cell virus (MSCV)-mCherry retroviral vector as described previously [36]. Human MCF-7, BT474 and SKBR3 lines were purchased from ATCC and cultured in DMEM supplemented with 10% foetal bovine serum (FBS), 2 mM l-glutamine, 1 mM sodium pyruvate and 1% penicillin-streptomycin. The metastatic MDA-MB-231 HM variant was provided by Dr. Yi-Feng Hou (Fudan University, Shanghai, China) and cultured in the same medium. For routine culture, cells were passaged when sub-confluent and kept in culture for a maximum of 4 weeks.

Lapatinib ditosylate, erlotinib hydrochloride, tucatinib hydrochloride, afatinib and RAS synthetic lethal 3 (RSL3) were obtained from SelleckChem (Scoresby, VIC, Australia). Erastin and liproxstatin-1 were purchased from Sigma-Aldrich (Castle Hill, NSW, Australia). Neratinib maleate was provided by Puma Biotechnology (Los Angeles, CA, USA). All compounds were prepared at 5–10 mM stocks in DMSO and diluted to the required concentration in the appropriate assay buffer immediately before in vitro and in vivo assays.

### In vitro proliferation and IC_50_ determination

Cell proliferation was measured using a sulforhodamine B (SRB) colorimetric assay as described previously with minor modifications [34, 37]. Briefly, TBCP-1 (1 × 10^3^) or SKBR3 (2 × 10^3^) cells were seeded in triplicate wells of a 96-well plate in 100 μl of serum-containing medium and allowed to adhere at 37 °C for 6 h. Inhibitors were added in 100 μl of the same medium and proliferation measured over 5 days. DMSO (≤ 1% *v*/*v*) was used as vehicle control. Half maximal inhibitory concentrations (IC_50_) values were determined in the same assay over 3 days with an initial cell density of 1 × 10^3^ (67NR, 4T1.2), 2 × 10^3^ (TBCP-1), 3 × 10^3^ (SKBR3) or 5 × 10^3^ (MCF-7, BT474, MDA-MB-231HM) cells/200 μl/well and IC_50_ values calculated using Hill’s equation in the GraphPad Prism 6.0 software. Where indicated, photographs were taken using an Olympus CKX53 inverted microscope to show evidence of cell death.

### Transcriptomic and bioinformatics analyses

Sub-confluent TBCP-1 cultures were incubated for 24 h in a serum-containing medium in the presence of neratinib (300 nM) or vehicle DMSO alone. RNA was extracted from replicate wells using the PureLink™ RNA isolation kit according to the manufacturer’s instructions (Thermo Fisher Scientific, Scoresby, VIC, Australia). All RNA samples were normalised to 1 μg for library preparation and then libraries normalised to 10 nM and pooled. Before sequencing, the pool was denatured with 0.2 N NaOH for 5 min at room temperature and the sample pool diluted to 1.8 pM and spiked with 2% PhiX. Sequencing was done on Illumina NextSeq 500 following the TruSeq Stranded mRNA Low Sample protocol according to the manufacturer’s instructions.

Raw reads quality control was performed by FastQC (v0.11.6). Adapter/primer sequences were clipped by trimgalore (v0.4.5). Cleaned reads were mapped to mouse genome (GRCm38.p5) by HiSat2 (v2.0.5) [38]. Alignment quality was checked with RSeQC (v2.6.4) [39]. The read counts for genes were computed using Subread (v1.4.6p5) [40] and then were subjected to edgeR (v 3.18.1) [41] for differential analysis. Genes were considered differentially expressed when the adjusted *p* value of the likelihood ratio was < 0.05. Functional enrichment analysis was carried out using goana and kegga function in EdgeR with adjustment for gene length.

### Immunoblotting

Expression of ERα, PR and HER2 in sub-confluent cultures of TBCP-1 cells was detected by standard immunoblotting [37]. Primary antibodies against ERα (Santa Cruz sc-542, 1 μg/ml), PR (Santa Cruz sc-538, 1 μg/ml) or HER2 (Abcam ab2428, 1 μg/ml) and appropriate horseradish peroxidase (HRP)-conjugated secondary antibodies were used to detect the respective proteins. An anti-GAPDH antibody (Abcam ab8245, 0.2 μg/ml) was used as a loading control.

For the expression of EGFR family of receptors and downstream signalling effectors, sub-confluent cultures were serum-starved overnight in serum-free medium supplemented with 1 mM sodium pyruvate, 2 mM glutamine and 1% penicillin/streptomycin and re-starved for 2 h in fresh serum-fee medium prior to exposure to neratinib for 1 h at 37 °C followed by the addition of EGF (100 ng/ml) (Thermo Fischer Scientific, #PHG0311) for 10 min at 37 °C. Cells were washed with ice-cold PBS and whole-cell lysates prepared in cell lysis buffer (30 mM HEPES, 5 mM EDTA, 150 mM NaCl, 1% (*v*/*v*) Triton X-100) supplemented with protease inhibitor cocktail (ROCHE, Sydney, NSW, Australia, #04693132001) and phosphatase inhibitor cocktail (Abcam, ab201112). Primary antibodies against EGFR (E235, Abcam, ab32077, 1/1000 dilution), phospho-EGFR (Y1173, Abcam ab5652, 1/1000 dilution), HER2 (ab2428, Abcam, 1/200 dilution), phospho-HER2 (Tyr877, Cell Signalling Technology, #2241, 1/1000 dilution), HER3 (ab5470, Abcam, 1/100 dilution), HER4 (E200, Abcam, ab 32375; 1/1000 dilution), MAPK (ERK1/2) (L34F12, Cell Signalling Technology, #4696, 1/1000 dilution), phospho-MAPK (Thr 202/Tyr204, Cell Signalling Technology, #9101, 1/1000 dilution), AKT (40D4, Cell Signalling Technology, #2920, 1/1000 dilution) and phospho-AKT (Ser 473, Cell Signalling Technology, #9271, 1/1000 dilution) were used to detect the respective proteins and specific binding detected using appropriate HRP-conjugated secondary antibodies and enhanced chemiluminescence (ECL) reagents (Amersham Biosciences, Castle Hill, NSW, Australia).

Ferroptosis, metabolic and apoptotic markers were analysed in whole-cell lysates from TBCP-1 sub-confluent cultures treated with DMSO (vehicle control) or neratinib (300 nM) or the BH3 mimetics ABT263 (0.5 μM) + MCL1 inhibitor S63845 (0.5 μM) for 6 h as indicated in the figure legend. Protein bands were detected with the following primary antibodies and appropriate HRP-conjugated secondary antibodies: Acyl-CoA synthetase long-chain family member 4 (ACSL4) (sc-271800, Santa Cruz Biotechnology, Santa Cruz, CA, USA, 1/1000 dilution), ferritin (ab75973, Abcam, 1/2000 dilution), transferrin receptor-1 (TFR-12-M, Alpha Diagnostics, San Antonio, TX, USA, 1/1000 dilution) and ferroportin-1 (NBP1-21502, Novus Biologicals, 1 μg/ml). Protein band intensity relative to GAPDH (Abcam ab8245, Abcam, 0.2 μg/ml) was quantitated using ImageJ software (National Institute of Health, Bethesda, MD, USA). For caspase 3 analysis (Cell Signalling Technology #9662, 1/1000 dilution), an anti-α-tubulin antibody (clone AA13, Sigma, 0.2 μg/ml) was used as a loading control.

### Inductively coupled plasma mass spectrometry

Sub-confluent cultures of TBCP-1 cells (5 replicates/condition) were treated for 72 h with vehicle alone (DMSO control) or neratinib (300 nM and 500 nM) and the cells pelleted by centrifugation. Fifty microlitres of concentrated nitric acid (65% *v*/*v*, Suprapur, Merck) was added to each cell pellet overnight at room temperature. Samples were heated at 90 °C for 20 min, and final volumes made up to 500 μl 1% (*v*/*v*) nitric acid. Iron content was measured using an Agilent 7700 series ICP-MS instrument under routine multi-element operating conditions in a Helium Reaction Gas Cell. The instrument was calibrated using 0, 5, 10, 50, 100 and 500 ppb of certified multi-element ICP-MS standard calibration solutions (ICP-MS-CAL2-1, ICP-MS-CAL-3 and ICP-MS-CAL-4, Accustandard) for a range of elements. A certified standard solution containing 200 ppb of yttrium (Y89) was used as an internal control (ICP-MS-IS-MIX1-1, Accustandard). The raw ppb results were normalised to final volume and converted to ng/10^6^ cell of metal using the formula: Final concentration (ng/10^6^ cell) = Raw ppb value (ng/mL) × sample volume (0.5 ml)/number of cells × 10^6^.

### Tumour growth, metastasis assays and neratinib therapy

All procedures involving mice conformed to the National Health and Medical Research Council animal ethics guidelines and were approved by the Austin Health Animal Ethics Committee (A2016/05346) and the Peter MacCallum Animal Ethics & Experimentation Committee (E507). Female BALB/C mice (5/box) were maintained in a specific pathogen-free environment with food and water freely available and monitored daily for signs of ill health or metastatic disease.

Experimental metastasis assays were done as described previously with minor modifications [34, 37]. For initial characterisation of metastatic spread, TBCP-1 cells (5 × 10^5^) were injected into the left cardiac ventricle of 6–8-week-old female BALB/C mice. The mice were monitored daily and sacrificed after 3 weeks or earlier if signs of metastases became apparent (weight loss > 10%, ruffled fur, lethargy, rapid breathing) (Additional file 1: Figure S1B). The organs were harvested, photographed, fixed in 10% buffered formalin for 24 h and processed for histology. Tumour growth and spontaneous metastasis assays were completed as described previously [34, 42]. For these assays, fluorescent mCherry-tagged TBCP-1 cells (1 × 10^6^/20 μl PBS) were inoculated into the fourth mammary fat pad and tumour growth measured thrice weekly with electronic callipers. Tumour volume was calculated using the formula (length × width^2^)/2. To document TBCP-1 distribution of spontaneous metastases, tumours were resected when they reached an average volume of ~ 400 mm^3^ (~ 3 weeks), and the experiment was terminated when the mice developed signs of metastatic disease (~ 7 weeks) (Additional file 1: Figure S1C). The incidence of mice with visible lung, liver, kidney, ovary and adrenal gland metastases was measured semi-quantitatively by visual examination at necropsy. For quantitation of metastasis incidence, the brains and bones were fixed for 24 h in 4% paraformaldehide (PFA) and paraffin-embedded. The bones were decalcified in 20% ethylenediaminetetraacetic acid (EDTA) prior to embedding. Three step-sections/organs (4 μm for bone or 6 μm for brain, 100 μm apart) were stained with haematoxylin and eosin (H&E) and the presence of brain or bone lesions confirmed on an Olympus BH2 phase contrast microscope.

The efficacy of neratinib was evaluated in the neoadjuvant or metastatic setting (Additional file 3: Figure S3B-3C). For neoadjuvant therapy, mice were treated daily with control vehicle [(0.5% (*w*/*v*) methylcellulose, 0.4% (*v*/*v*) polysorbate-80] or neratinib (60 mg/kg) by oral gavage, starting when mammary tumours reached ~ 100 mm^3^ (~ 1 week). Treatment was stopped and tumours resected when control tumours reached ~ 400–500 mm^3^ or after a maximum of 3 weeks. In the experimental metastasis setting, treatment commenced 2 days after the intra-cardiac inoculation of the cells, and ceased after a maximum of 3 weeks, or earlier if mice showed signs of advanced metastatic disease. Mice were sacrificed individually when they developed signs of advanced metastatic disease, and survival was plotted by Kaplan-Meier analysis. The incidence of mice with brain metastases at endpoint was determined by ex vivo fluorescence imaging using a Maestro™ In-Vivo Imaging System (CRi) and, where indicated, further confirmed by histology and cytokeratin staining (see below). Relative metastatic burden in the lungs, femurs and spine of the control and treatment groups was quantitated by genomic qPCR detection of the mCherry marker gene present in tumour cells only, relative to the vimentin gene present in tumour and host cells, as described previously [34, 37, 42].

### Histology and immunohistochemical staining

Tissues were fixed in 10% buffered formalin for 24 h and processed for paraffin embedding. The sections (4 μm for the bone or 6 μm for the brain) were cut using a Leica RM 2245 Microtome and metastatic lesions in the bones or brains identified by standard haematoxylin and eosin (H&E) staining. Epithelial (cytokeratin), proliferative (Ki-67) ER, PR and HER2 status were assessed by standard IHC staining as described [34, 43]. Primary antibody for pan-cytokeratin (Sigma C801, 2.5 μg/ml), Ki-67 (Millipore AB9260, 2 μg/ml), ERα (Santa Cruz sc-542, 1 μg/ml), PR (Santa Cruz sc-538, 1 μg/ml) and HER2 (Calbiochem 3B5, 10 μg/ml) and appropriate biotin-conjugated secondary antibodies were used to detect the respective proteins. Staining was visualised using a 3,3′-diaminobenzidine (DAB) liquid substrate system (Sigma-Aldrich). Quantitative scoring of IHC staining was done on scanned whole tumour and brain sections (*n* = 3) using Aperio ImageScope software v11.1.2.760. For ERα, PR and Ki-67 scoring, the data are expressed as percentage of positive nuclei in whole tumours (excluding necrotic areas) or in delineated cytokeratin^+ve^ brain lesions. Pan-cytokeratin was scored as percentage of positive cells (membrane and/or cytoplasmic expression). For HER2 quantitation, the sections were scored as 3+ for strong continuous membrane expression, 2+ for moderate continuous membrane expression and 1+/0 for weak/discontinuous or no expression.

### Statistical methods

Data were analysed for statistical significance using GraphPad Prism 6 software. Unless otherwise indicated, data from in vitro experiments are presented as mean ± SD and data from in vivo experiments are presented as mean ± SEM. Statistical significance between the two groups was analysed by Mann-Whitney, non-parametric test. Experiments with more than two groups were analysed with one-way analysis of variance (ANOVA) and Tukey’s multiple comparison test. Statistical analysis of tumour volume was assessed by two-way ANOVA and Bonferroni’s post-test. Survival proportions were determined using Kaplan-Meier analysis and further analysed with a Gehan-Breslow-Wilcoxon test. Incidence data were analysed by Fisher’s exact test. *p* < 0.05 was considered significant.

## Results

### Development and characterisation of the TBCP-1 model

**Fig. 1:**
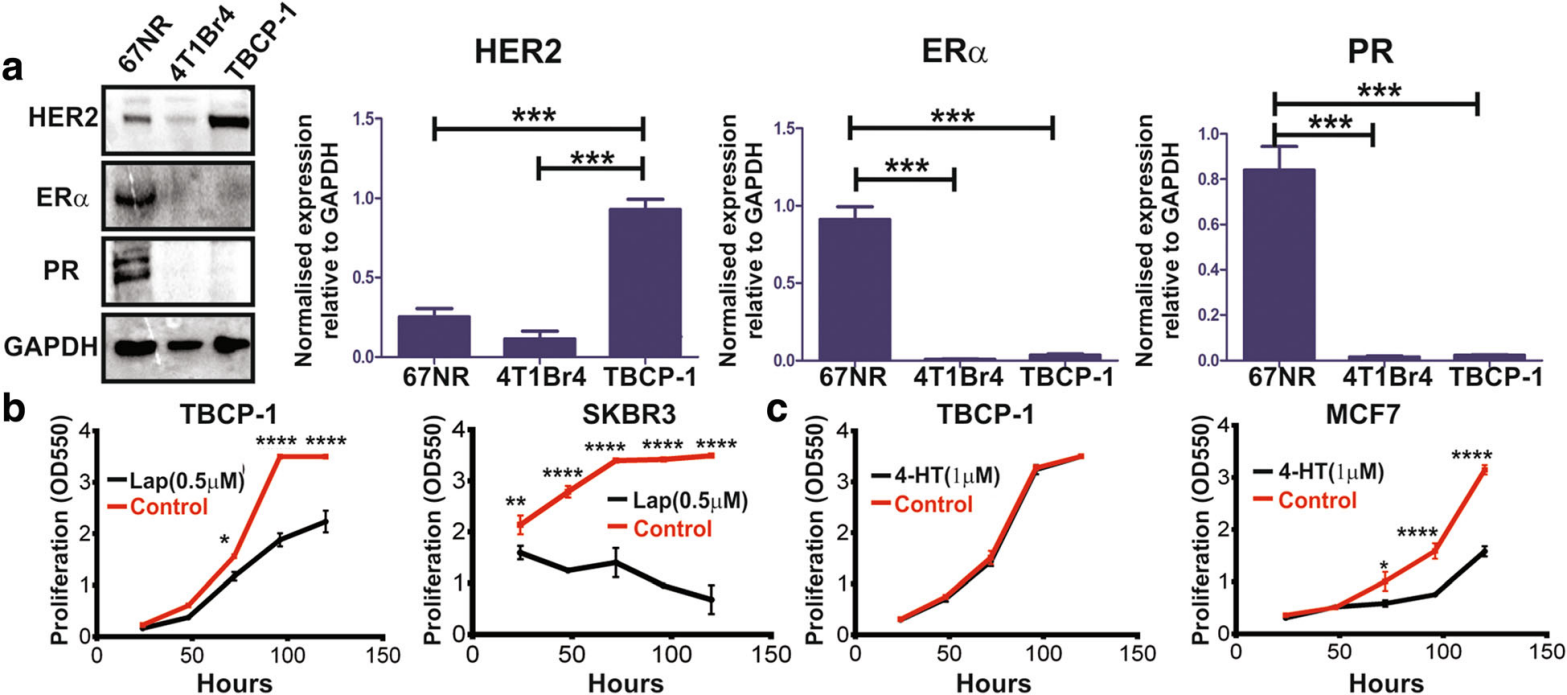
TBCP-1 cells are phenotypically and functionally similar to HER2^+ve^ breast cancer. **a** Representative western blots (left panel) and quantitation (right panels) of ERα, PR and HER2 protein expression. Data in the right panels show relative protein expression normalised to GAPDH and are expressed as mean ± SD of three independent experiments (*n* = 3). 67NR cells were used as a positive control for ER and PR, and 4T1Br4 used as a negative control for all three receptors. ***p < 0.001. TBCP-1 cell proliferation in the presence of **b** lapatinib or **c** 4-hydroxytamoxifen was measured over 5 days in a standard SRB proliferation assay. SKBR3 and MCF7 were used as HER2- or ER-positive controls, respectively. Each point on the curves represents the mean ± SD of three independent experiments (*n* = 3). *p < 0.05, ****p < 0.0001

Parental TBCP-1 cells were derived from the spontaneous BALB/C mammary tumour-1 (SMT1) and initially grew slowly in culture. The cells were gradually adapted to standard in vitro culture over several months (Additional file 1: Figure S1A). Clonal populations were isolated from the morphologically heterogeneous parental TBCP-1 cells by fluorescence-activated cell sorting (FACS) (Additional file 1: Figure S1A) and further evaluated for the expression of hormone (ERα, PR) and HER2 receptors by immunoblotting and for metastatic ability in vivo. A clone naturally expressing high levels of membrane HER2, but not ER or PR protein (hereafter referred to as TBCP-1), was selected for its phenotypic similarity to the HER2^+ve^ subtype of breast cancer (Fig. 1a, Additional file 1: Figure S1A). By comparison, luminal-like 67NR cells showed high expression of ERα and PR but very low levels of HER2 whereas the brain metastatic triple-negative 4T1Br4 line [34] lacked the expression of ERα and PR receptors and showed only modest expression of HER2. The ER^-ve^/PR^-ve^/HER2^+ve^ phenotype of TBCP-1 cells was functionally relevant since their proliferation was significantly reduced by the HER2 inhibitor lapatinib (Fig. 1b), but not by the ER modulator 4-hydroxytamoxifen (Fig. 1c).

### TBCP-1 tumours metastasise avidly to the brain, bone and soft tissues

**Fig. 2:**
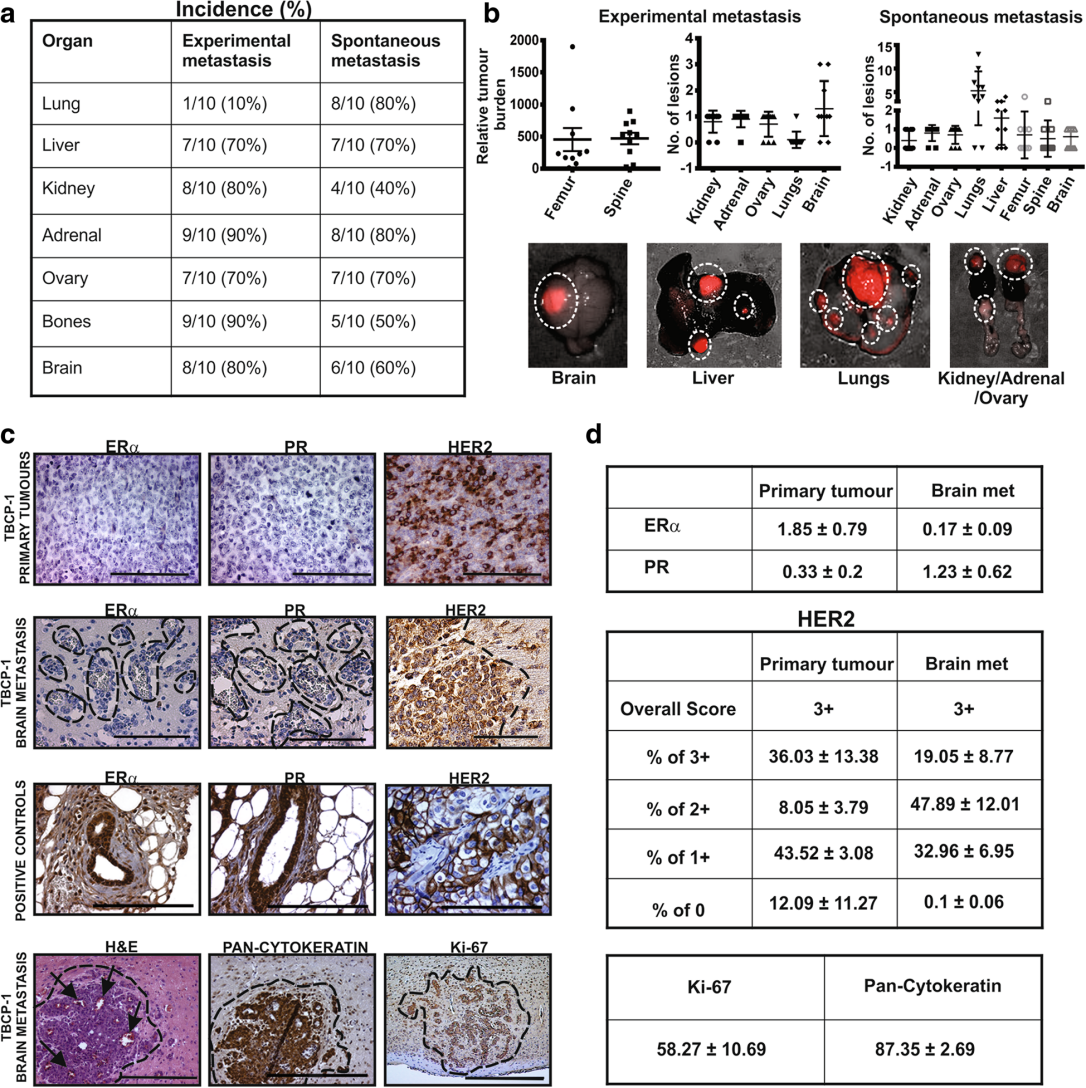
TBCP-1 tumours metastasise avidly to the brain and other organs. **a** Summary of the incidence of mice developing experimental or spontaneous metastases after intra-cardiac or orthotopic inoculation of TBCP-1 cells. **b** Quantitation of tumour burden and number of metastatic lesions/organ from experimental and spontaneous metastasis assays. Experimental femur and spine metastatic burden was quantitated by genomic qPCR of the mCherry reporter gene as described in the “Methods” section. Each dot represents one mouse, and data show mean ± SEM, *n* = 10. The number of lesions was determined by visual examination of histological sections. Each dot represents one mouse, and data show mean ± SD, *n* = 10. Representative images of soft organ metastases (red, with brightfield overlay) that developed after orthotopic inoculation of TBCP-1 cells are shown in the bottom panels. Lesions are delineated by a dotted line. **c** ERα, PR and HER2 were detected in TBCP-1 primary tumours or brain metastases by standard immunohistochemistry as indicated. Nuclear ERα and PR detected in normal mammary gland surrounded by a 67NR primary tumour and membrane HER2 detected in a SKBR3 primary tumour were used as positive controls (third row). H&E staining (arrows = blood vessels), pan-cytokeratin and Ki-67 positivity in TBCP-1 brain metastases are shown (bottom panels). Dotted lines delineate metastatic lesions. Scale bars = 100 μm. **d** Quantitation of ERα, PR, HER2, Ki-67 and pan-cytokeratin protein levels in primary tumours and brain metastases. Scoring of tissue sections was done using the ImageScope software as described in the “Methods” section. Data show mean ± SEM of three independent tumours/metastases

Breast cancer metastasis models are broadly categorised as experimental or spontaneous depending on the route of tumour cell inoculation, i.e. intra-vascular or mammary fat pad, respectively. To assess the intrinsic metastatic properties of TBCP-1 cells in vivo, metastatic spread in BALB/C mice was first documented in an experimental intra-cardiac inoculation model, and metastasis scored semi-quantitatively by visual inspection at necropsy when the mice developed signs of metastatic disease (Additional file 1: Figure S1B). Intra-cardiac injection is commonly used to investigate CNS metastasis, as the vascular flow facilitates dissemination of tumour cells to the brain, bone, ovaries and adrenal glands, even in tumour lines that are weakly metastatic from the mammary gland [44]. Consistent with these observations, this protocol gave rise to a low incidence of lung metastases but high incidence of visible macro-metastases in the liver, kidneys, ovaries, adrenal glands and bone (Fig. 2a, b). Genomic qPCR of the mCherry reporter gene further confirmed the presence of femur and/or spine metastases in 90% of animals (Fig. 2a, b). Importantly, H&E and cytokeratin staining of the paraffin sections revealed that 80% of the mice developed brain metastases (Fig. 2a, b).

To recapitulate the complete metastatic cascade, TBCP-1 cells were injected into the fourth inguinal fat pad of BALB/C mice and primary tumours were resected after 3 weeks when they reached an average size of ~ 400 mm^3^ to prevent early termination of experiment due to unethical primary tumour size and to more closely mimic the clinical scenario (Additional file 1: Figure S1C). The mice were sacrificed when signs of metastatic disease were evident and metastatic spread documented. Widespread spontaneous metastasis to the soft organs and bone was observed (Fig. 2a, b). As expected, the incidence of spontaneous lung metastases was higher (80%) than experimental lung metastases (10%). Conversely, spontaneous bone metastases using this protocol were lower (50%) than in the experimental metastasis model (90%). Remarkably, H&E and cytokeratin staining of the brain sections indicated that ≥ 60% of the mice developed spontaneous brain metastases, generally limited to a single large lesion compared to multiple mCherry-positive nodules detected in lungs and liver (Fig. 2a, b).

Importantly, IHC staining confirmed that TBCP-1 primary tumours and brain metastases maintained their hormone receptor negativity and strong HER2 protein expression in vivo (Fig. 2c). Quantitative scoring of ERα and PR expression (% of positive nuclei) in three independent primary tumours and brain metastases showed that nuclear expression of these receptors is negligible (Fig. 2d). In contrast, HER2 positivity was heterogeneous but consistent with a HER2^+ve^ phenotype, as defined by high continuous membrane staining (3+) in > 10% of the cells (Fig. 2d). In addition, TBCP-1 brain metastases were highly vascularised, expressed cytokeratins and were highly proliferative, evidenced by Ki-67 positivity (Fig. 2c, d). Collectively, these results indicate that TBCP-1 tumours are highly metastatic to multiple organs, including to the brain, in immune-competent mice and are phenotypically similar to the HER2^+ve^ subtype of breast cancer.

### Evaluation of TBCP-1 response to TKIs in vitro

**Fig. 3:**
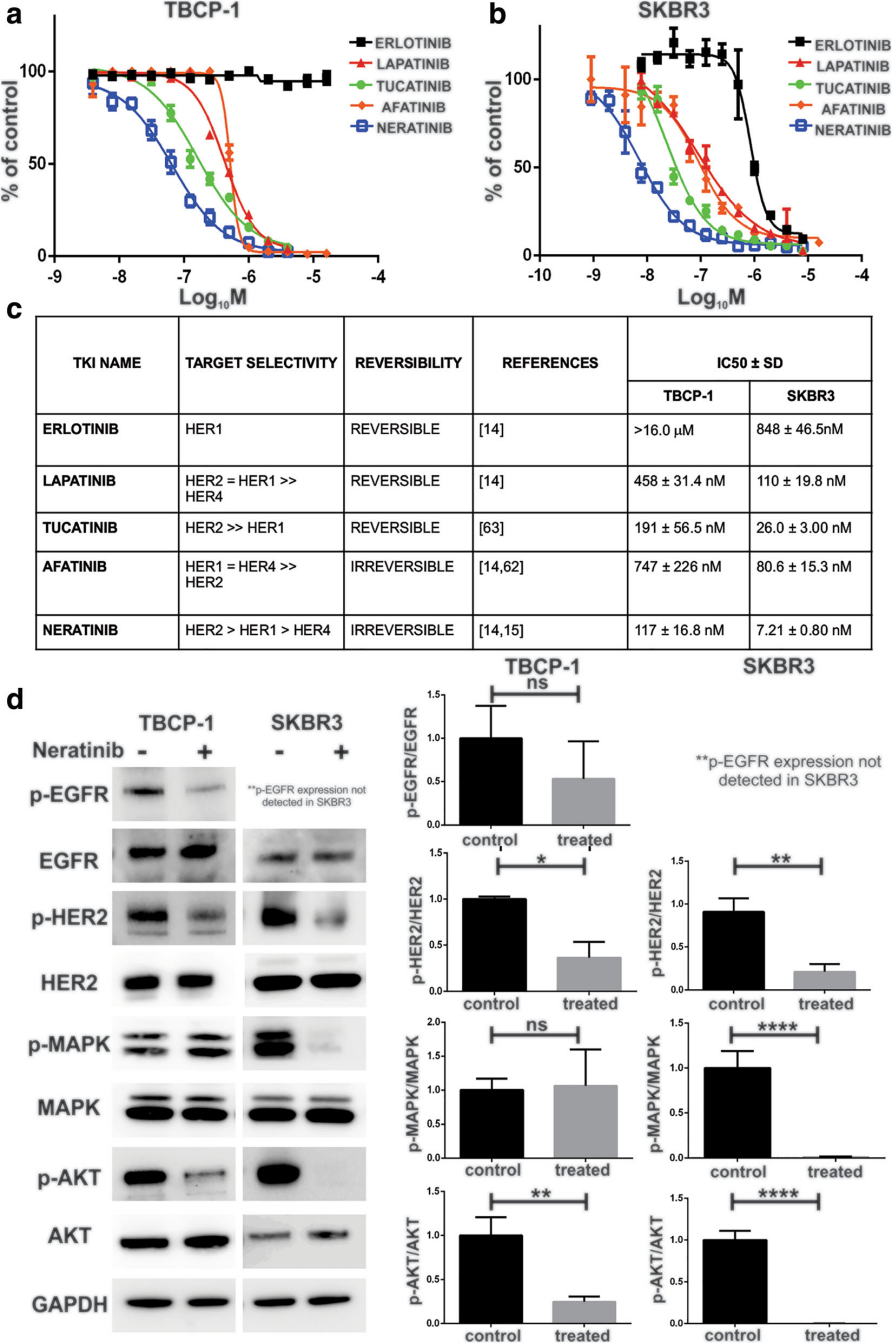
Neratinib inhibits cell proliferation and HER2 signalling in vitro. Proliferation of **a** TBCP-1 and **b** SKBR3 cells was measured over 3 days in the presence of TKIs in a standard 3-day SRB colorimetric assay. Dose-response curves were plotted to determine the half-maximal inhibitory concentrations (IC_50_) for each compound using Hill’s equation in the GraphPad Prism software. Each point on the curves represents the mean ± SD of three independent experiments performed in triplicate (*n* = 3). **c** Summary of TKI receptor selectivity and IC_50_ in TBCP-1 and SKBR3 cells. **d** Changes in levels of p-EGFR, total EGFR, p-HER2, total HER2, p-MAPK, total MAPK, p-AKT and total AKT proteins in TBCP-1 or SKBR3 cells following 1 h of neratinib treatment (300 nM for TBCP-1 or 5 nM for SKBR3) were measured by western blotting. Representative blots are shown on the left. GAPDH was used as a loading control. Densitometric quantitation of phospho/total protein ratio (right panels) was completed using ImageJ software. Data show mean ± SD of three independent experiments (*n* = 3). *p < 0.05, **p < 0.01, ****p < 0.0001

Next, we compared the sensitivity of mouse TBCP-1 and human SKBR3 HER2^+ve^ cells to a panel of TKIs in a short-term (3-day) proliferation assay (Fig. 3a, b). The reversible EGFR inhibitor, erlotinib, did not significantly inhibit the proliferation of TBCP-1 cells (IC_50_ > 16 μM) but inhibited SKBR3 cells with an IC_50_ of 848 nM. As expected, the dual EGFR/HER2-reversible inhibitor lapatinib and the selective HER2-reversible inhibitor tucatinib inhibited TBCP-1 cells (IC_50_, 458 nM and 191 nM, respectively) albeit at concentrations higher than in SKBR3 cells (IC_50_, 110 nM and 26 nM, respectively) known to be highly sensitive to TKI inhibition [16, 45]. Both lines were also sensitive to the irreversible pan-inhibitor afatinib but with higher IC_50_ values of 747 nM (TBCP-1) and 80.6 nM (SKBR3). The irreversible pan-inhibitor, neratinib, was the most potent TKI against both cell lines in this assay, inhibiting TBCP-1 and SKBR3 cells with IC_50_ of 117 nM and 7.2 nM, respectively. The results for each TKI and their receptor selectivity are summarised in Fig. 3c.

In preliminary experiments, we confirmed that TBCP-1 and SKBR3 cells express HER1, HER2 and HER3 but not HER4 (Additional file 1: Figure S1A). Thus, the impact of neratinib on activation of HER1 (EGFR) and HER2 and downstream signalling in vitro was investigated by immunoblotting (Fig. 3d). For these experiments, cells were serum-starved and treated with neratinib for 1 h prior to stimulation with EGF for 10 min. Under those conditions, neratinib only partially reduced EGF-induced phosphorylation of EGFR but significantly inhibited HER2 activation in TBCP-1 cells. In SKBR3 cells, neratinib almost completely inhibited HER2 and p-EGFR was undetectable. Surprisingly, while phosphorylation of the downstream target, AKT, was also significantly inhibited by neratinib in both lines, MAPK phosphorylation (p-ERK-1/2) was inhibited in SKBR3 but not in TBCP-1 cells.

### Neratinib induces cell death by ferroptosis

The mechanisms that could contribute to the superior inhibitory activity of neratinib were further investigated by profiling the transcriptome of TBCP-1 cells cultured in the presence or absence of neratinib (0.3 μM) for 24 h, when early morphological changes (rounding), prior to cell death, were evident (Additional file 2: Figure S2A). Consistent with their response to neratinib, principal component analyses showed a clear differential clustering of untreated and neratinib-treated TBCP-1 cells (Additional file 2: Figure S2B). RNA-seq profiling identified a total of 407 genes that were differentially expressed (DEGs) in untreated versus neratinib-treated TBCP-1 cells (false discovery rate (FDR) < 0.05; LogFC > 1) including 206 upregulated genes and 201 downregulated genes. Gene ontology analysis of upregulated genes in neratinib-treated TBCP-1 cells identified significant enrichment in GO terms related to metabolic and biosynthetic processes (Additional file 4: Table S1). Genes upregulated in response to neratinib were also enriched in multiple KEGG pathways including those associated with MAPK and PIK3-Akt signalling pathways, EGFR tyrosine kinase inhibitor resistance and ferroptosis (Additional file 5: Table S2).

**Fig. 4:**
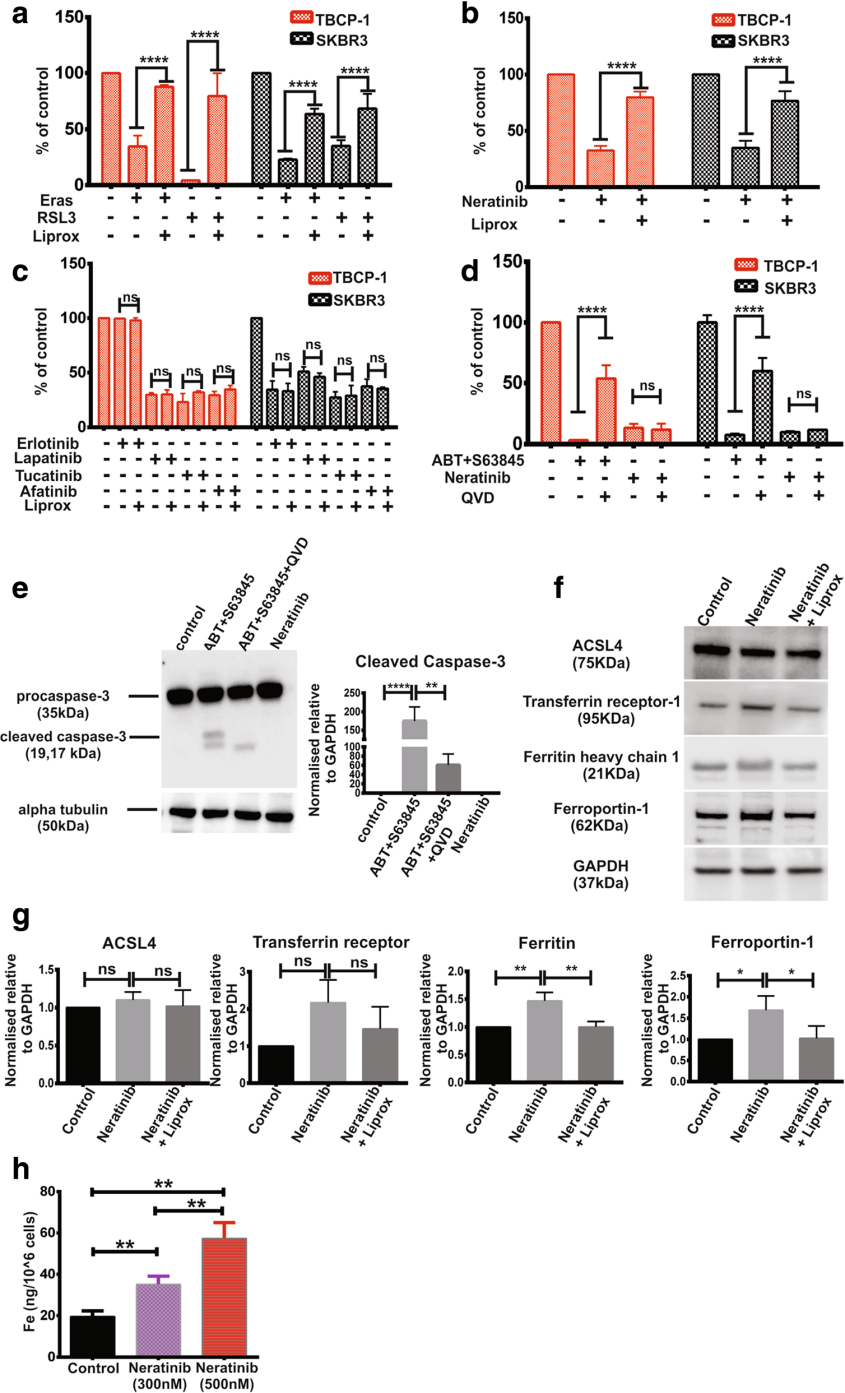
Neratinib induces cell death by ferroptosis. **a** Liproxstatin-1 prevents erastin- or RSL3-induced ferroptotic cell death in TBCP-1 (red bars) or SKBR3 cells (black bars). **b** Neratinib-induced cell death in TBCP-1 or SKBR3 is rescued by co-incubation with liproxstatin-1. **c** Erlotinib-, lapatinib-, afatinib- and tucatinib-induced TBCP-1 (red bars) or SKBR3 (black bars) cell death is not rescued by liproxstatin-1. Cells in **a** and **b** were incubated with erastin (5 μM), RSL3 (0.5 μM) or neratinib (300 nM, TBCP-1 or 5 nM, SKBR3) with or without liproxstatin-1 (2 μM) as indicated. TKIs in **c** were used at the following concentrations based on their relative activity determined in Fig. 3a and b for TBCP-1 and SKBR3, respectively; erlotinib (16 μM and 500 nM), lapatinib (500 nM and 100 nM), tucatinib (300 nM and 50 nM) and afatinib (500 nM and 100 nM). Viable proliferating cells were quantitated after 72 h by SRB staining. Data in **a**–**c** are expressed as percentage of control vehicle-treated cells and show mean ± SD of three independent experiments performed in triplicates. ****p < 0.0001. n/s, not significant. **d** Neratinib does not induce caspase-dependent apoptosis. Cells were treated for 6 h with a combination of BH3 mimetic (ABT263, 0.5 μM) + MCL1 inhibitor (S63845, 0.5 μM) or with neratinib (300 nM) in the absence or presence of caspase inhibitor Q-VD (20 μM). **e** Whole-cell lysates from control (DMSO), BH3 mimetics or neratinib-treated TBCP-1 cells were analysed by western blotting for the expression of caspase-3. A representative blot from three independent experiments is shown on the left and quantitation of cleaved caspase-3 bands relative to α-tubulin is shown on the right (*n* = 3). **p < 0.01, ***p < 0.001. **f**, **g** Lysates from control, neratinib or neratinib + liproxstatin-treated cells were analysed for the expression of ACSL4, ferritin, ferroportin-1 and transferrin receptor-1. A representative blot from three experiments is shown in **f** and quantitation relative toGAPDH (*n* = 3) shown in **g**. *p < 0.05, **p < 0.01. **h** ICP-MS analysis of iron content in control and inhibitor-treated TBCP-1 cells. Data are expressed as ng/10^6^ cells and show mean ± SD of six replicate samples. **p < 0.01

Ferroptosis is a recently described form of cell death distinct from apoptosis and characterised by iron and reactive oxygen species (ROS)-dependent oxidative stress leading to excessive lipid peroxidation that compromises the integrity of cell membranes [46–49]. The clinically approved TKI, sorafenib, has been shown previously to induce ferroptosis [50]. However, whether neratinib induces ferroptosis has not been reported. To determine if TBCP-1 or SKBR3 cells are susceptible to ferroptotic death, cells were treated with potent ferroptosis inducers, erastin or RSL3 [51, 52]. Both compounds induced ferroptosis in TBCP-1 or SKBR3 cells, and this response was rescued by liproxstatin-1, an inhibitor of ferroptosis [53] (Fig. 4a). Importantly, neratinib-induced cell death was also prevented by liproxstatin-1, demonstrating for the first time that neratinib acts in part by inducing ferroptosis (Fig. 4b, Additional file 2: Figure S2C). To address whether this property was specific to neratinib or shared with other TKIs, we asked if the inhibitory activity of erlotinib, lapatinib, tucatinib and afatinib could be blocked by treatment with liproxstatin-1 (Fig. 4c). Unlike neratinib, the inhibitory effect of these TKIs on TBCP-1 and SKBR3 cells was not prevented by liproxstatin-1.

Ferroptosis is distinct from apoptosis, as it does not require caspase activation [54]. Thus, further experiments were completed to distinguish these forms of cell death following neratinib treatment. Apoptosis was strongly induced in TBCP-1 and SKBR3 cells by a combination of two BH3 mimetics, (ABT263) and the MCL1-selective inhibitor (S63845) (Fig. 4d) and accompanied by extensive membrane blebbing, a classical feature of apoptosis (Additional file 2: Figure S2C). These responses were significantly prevented by treatment with the caspase inhibitor Q-VD. In contrast, Q-VD did not prevent neratinib-induced cell death (Fig. 4d, Additional file 2: Figure S2C). The differential apoptotic response was also evidenced by activation/cleavage of caspase 3 by ABT263/S63845 and partial rescue by Q-VD, but not by neratinib (Fig. 4e).

To further validate the pro-ferroptotic activity of neratinib, we examined the expression of selected ferroptosis regulators identified through KEGG pathway analyses (Additional file 6: Table S3). ACSL4, a key regulator of fatty acid metabolism and biomarker of ferroptosis, catalyses the production of 5-hydroxyeicosatetraenoic acid [55, 56]. Its expression in TBCP-1 cells was not significantly induced by treatment with neratinib (Fig. 4f, g). Ferritin, a regulator of iron metabolism and the main cellular iron storage protein, captures free Fe^2+^ to maintain iron homeostasis [57]. Ferritin levels in TBCP-1 cells increased ~ 1.5-fold in response to neratinib treatment. Transferrin receptor-1, the main cellular iron (Fe^3+^) importer, increased ~ 2-fold after exposure to neratinib, but this response was variable and did not reach statistical significance. Ferroportin-1 is required for the export of intracellular iron [58]. In agreement with the levels reported in breast cancer lines [59], its level was very low in control but increased ~ 1.6-fold in response to neratinib. Increased ferritin and ferroportin-1 levels most likely reflect an initial attempt (but eventual failure) by the cells to maintain iron homeostasis following neratinib treatment. Consistent with this, ICP-MS analysis at 72 h revealed a dose-dependent increase in the intracellular concentration of iron (Fig. 4h). Taken together, these results demonstrate conclusively the pro-ferroptotic activity of neratinib in HER2^+ve^ breast cancer cells.

To gain further insight on the relationship between neratinib-induced ferroptosis and breast cancer subtypes, we examined the sensitivity of other mouse and human lines to neratinib or RSL3 in the presence or absence of liproxstatin-1 (Additional file 3: Figure S3A). Murine luminal-like 67NR cells and 4T1.2 (TNBC) showed low sensitivity to neratinib (IC_50_ = 0.638 μM and 2.383 μM, respectively) but only inhibition by RSL3 (0.5 μM) was rescued by liproxstatin-1. Amongst the human lines, HER2^+ve^ BT474 expressed very high levels of HER2 and was highly sensitive to neratinib (IC_50_ ~ 1 nM) or RSL3. The effect of neratinib (2 nM) or RSL3 (0.5 μM) was rescued by liproxstatin-1. The luminal MCF-7 line expressed low levels of HER2 but not EGFR and showed poor sensitivity to neratinib (IC_50_ > 4 μM). These cells were susceptible to RSL3-induced ferroptosis but inhibition by neratinib, even at high dose (5 μM), was not rescued by liproxstatin-1. The TNBC line, MDA-MB-231HM, was inhibited by either neratinib used at 0.5 μM (IC_50_ = 0.259 μM) or RSL3, but only the latter was rescued by liproxstatin-1. MDA-MB-231HM cells expressed EGFR but very low levels of HER2 suggesting that the effect of neratinib in these cells is most likely mediated through EGFR rather than HER2 inhibition (Additional file 3: Figure S3A).

### Evaluation of neratinib response in vivo

**Fig. 5:**
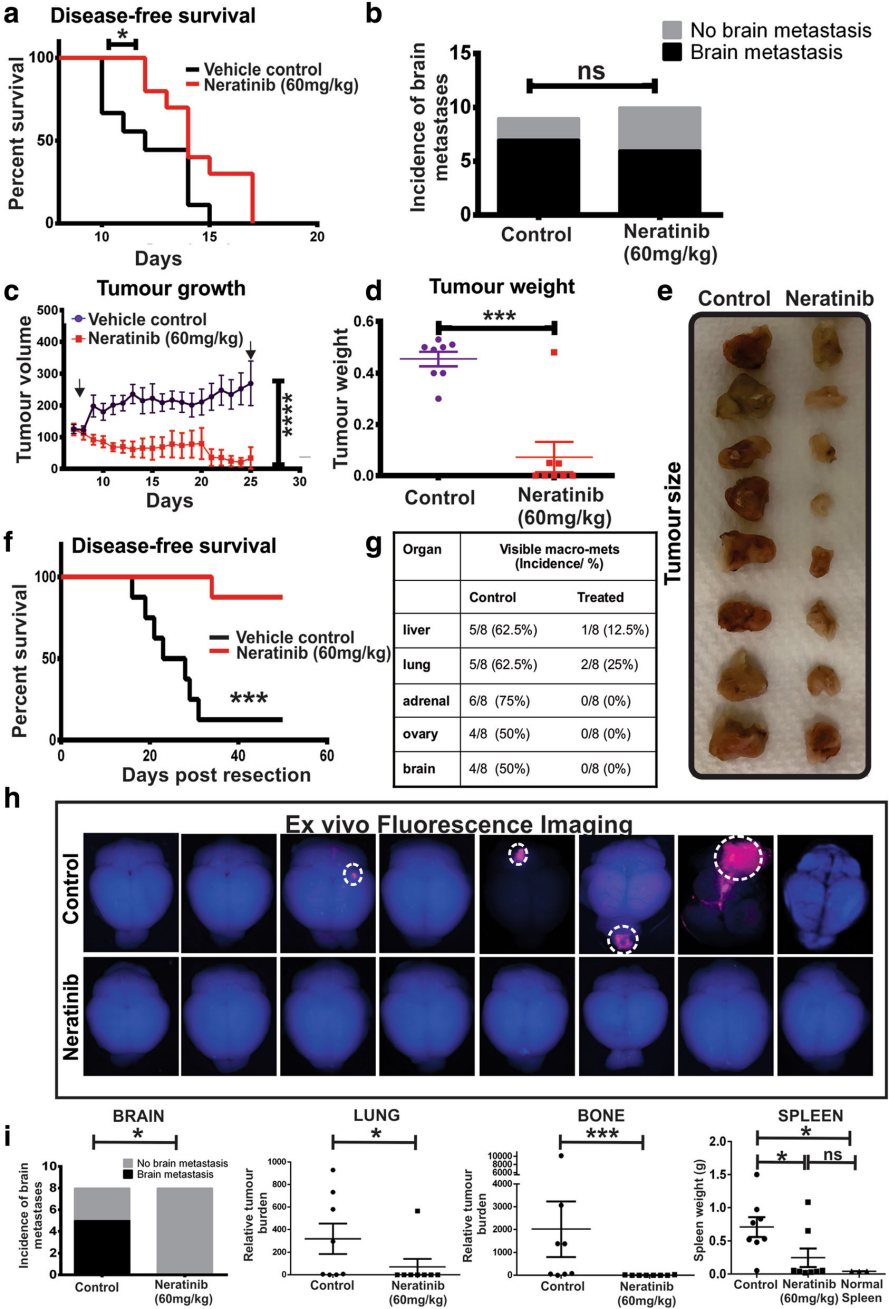
Neoadjuvant neratinib prolongs survival and inhibits metastasis more potently than late intervention. **a** Kaplan-Meier survival analysis of control (vehicle) and neratinib-treated (60 mg/kg) mice. TBCP-1 cells were inoculated into the left cardiac ventricle and the mice treated daily by oral gavage starting 2 days after tumour cell inoculation. Control, *n* = 9; neratinib, *n* = 10. *p = 0.0424. **b** Incidence of mice with visible brain metastases was determined by screening of three H&E step-sections/brain (100 μm apart) from the control and neratinib-treated groups. n/s, not significant. **c**–**h** Orthotopic tumour-bearing mice (8/group) were treated daily with vehicle (control) or neratinib (60 mg/kg) by oral gavage for up to 3 weeks, starting when tumours reached 100 mm^3^ and surgically resected on day 25, as described in the “Methods” section. **c** Tumour growth rate. Data show mean ± SEM. ****p < 0.0001. Arrows show start/end of treatment. **d** Tumour weight at resection. Each dot represents one mouse. Data show mean tumour weight ± SEM. ****p < 0.0001. **e** Image of primary tumours from control and neratinib-treated mice. **f** Kaplan-Meier survival analysis. ***p < 0.00001. **g** Incidence of soft tissue macro-metastases at endpoint in vehicle control and neratinib-treated mice. **h** Ex vivo fluorescence imaging (mCherry) of the brains at endpoint. Macro-metastases (mCherry^+ve^) are delineated by a dotted line. **i** Incidence of mice with visible brain micro-/macro-metastases (left panel) was determined by screening of three H&E step-sections/brain (100 μm apart) from control and neratinib-treated mice (*n* = 8) and further confirmed by cytokeratin IHC staining. *p < 0.05. Metastatic burden in the lung and bone (combine matched femur and spine burden) (middle panels) was determined by genomic qPCR of the mCherry marker gene (tumour cells) relative to vimentin (tumour + host cells) as described in the “Methods” section. Data are expressed as relative tumour burden (RTB) and show one point per mouse and mean burden (horizontal bar) ± SEM (*n* = 8/group). *p < 0.05, **p < 0.01, ***p < 0.001. Spleen weight in naïve (*n* = 3), vehicle control (*n* = 8) and neratinib-treated (*n* = 8) mice were measured at endpoint. Each point represents one mouse, and data show mean spleen weight ± SEM

The efficacy of neratinib on late-stage metastatic disease was evaluated first in an experimental metastasis assay in which the cells were inoculated directly into the left cardiac ventricle to bypass the formation of a primary tumour, a protocol commonly used to promote brain and bone metastasis [44] (Additional file 3: Figure S3B). Compared to control mice, there was a modest but statistically significant increase in disease-free survival in mice treated with neratinib (60 mg/kg) (Fig. 5a), an effective dose [15] that was well tolerated and did not induce weight loss or diarrhoea in BALB/C mice. Histological examination of the brains at endpoint showed a minor decrease in the incidence of mice with detectable brain lesions (6/10, 60%) compared to control (7/9, 78%) although this difference was not statistically significant (*p* = 0.6285) (Fig. 5b).

We next tested the efficacy of neratinib neoadjuvant monotherapy, with neratinib (60 mg/kg) given daily for 3 weeks, commencing when primary tumours reached ~ 100 mm^3^ and after which primary tumours were surgically resected (Additional file 3: Figure S3C) Under those conditions, neratinib dramatically reduced tumour growth (Fig. 5c), with several tumours almost completely regressing over the treatment period. This was further confirmed by a significant difference in tumour weight and size at resection (Fig. 5d, e). Importantly, disease-free survival of neratinib-treated mice was dramatically prolonged compared to control mice (Fig. 5f). Moreover, a significant reduction in the incidence of visible mCherry^+ve^ macrometastases in several organs including the liver, lung, adrenal gland and ovaries was observed in neratinib-treated mice compared to vehicle-treated mice (Fig. 5g). Only small tumour nodules were found in the liver of one mouse and in the lung of two mice in the treatment group (Fig. 5g). Remarkably, mCherry^+ve^ brain macro-metastases were detected in half of the control mice, but none were visible in the neratinib-treated group (Fig. 5g, h). Examination of brain step-sections from control and neratinib-treated mice identified one additional mouse with micro-metastases in the control group but not in mice treated with neratinib (62.5% versus 0%) (Fig. 5i). The metastatic burden in the lung and bone was further quantitated by genomic qPCR of the mCherry gene and found to be significantly decreased in neratinib-treated mice (Fig. 5i). Consistent with the immune-competent nature of the TBCP-1 model, high metastatic burden in control mice was associated with a significant enlargement of the spleen, a characteristic of syngeneic metastasis models such as murine 4T1-derived models that generally correlates with overall disease burden [34] (Fig. 5i). Importantly, spleen weights were dramatically reduced in neratinib-treated mice.

**Fig. 6:**
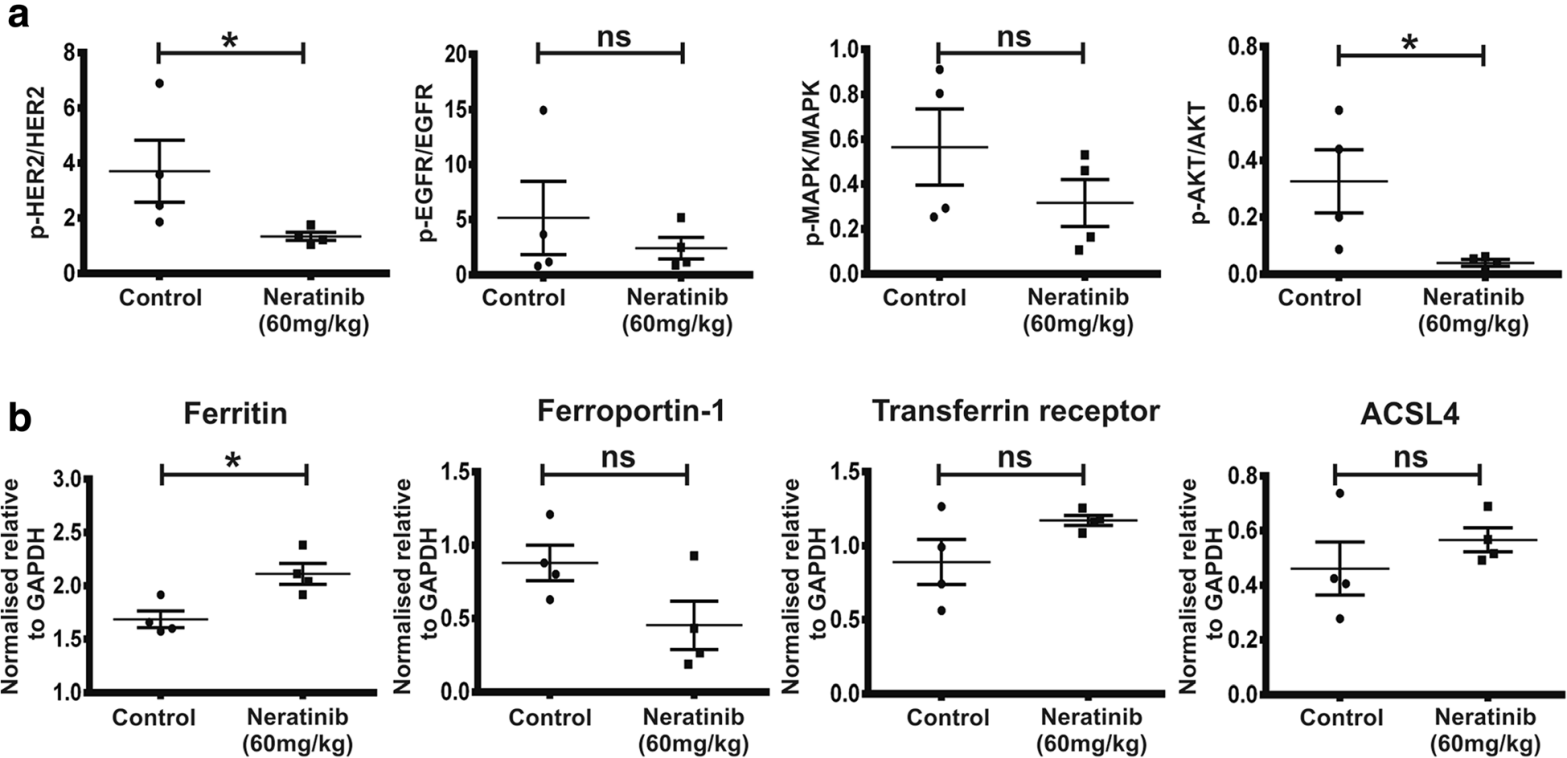
Impact of neratinib on HER2 signalling and iron metabolism in TBCP-1 tumours. Mice with palpable tumours were treated daily with vehicle or neratinib (60 mg/kg) for 4 days and tumours resected 2 h after the last neratinib dose. **a** Tumour lysates were analysed for the expression of total or phosphorylated EGFR, HER2, MAPK and AKT by western blotting as indicated and the protein bands quantitated by densitometry as described in the “Methods” section. The results are expressed as the ratio of phospho/total protein. **b** Markers of ferroptosis/iron metabolism were analysed by western blotting as described above and band intensity normalised to GAPDH. Each point in **a** and **b** represents one mouse and data show mean ± SEM of four control or neratinib-treated tumours (*n* = 4). *p < 0.05

In a separate experiment, EGFR and HER2 receptor status and downstream signalling intermediates were also analysed in primary tumours from control and neratinib-treated mice to corroborate our in vitro observations. For these experiments, mice with palpable tumours were treated daily with vehicle or neratinib (60 mg/kg) for 4 days and tumours resected 2 h after the last neratinib dose, when reduced growth rate and/or the beginning of regression was evident. Consistent with in vitro observations (Fig. 3d), we found that HER2 and Akt phosphorylation decreased significantly in TBCP-1 primary tumours upon neratinib treatment (Fig. 6a). A trend towards decreased EGFR and ERK-1/2 phosphorylation was also observed but did not reach statistical significance. Further analysis of markers of iron metabolism/ferroptosis in TBCP-1 tumours revealed increased levels of ferritin (*p* < 0.05) and a strong trend towards decreased ferroportin-1. Transferrin receptor-1 and ACSL4 increased slightly in the neratinib-treated group but were not significantly different from control tumours (Fig. 6b). Collectively, these observations demonstrate that first-line neoadjuvant neratinib therapy potently inhibits orthotopic HER2^+ve^ tumour growth and dramatically reduces the incidence of metastases in the brain and other organs in immune-competent BALB/C mice, resulting in a significant improvement in disease-free survival. These responses are accompanied by attenuation of HER2 receptor signalling and alteration in iron metabolism associated with ferroptosis.

## Discussion

TKIs are increasingly used in the clinic for the treatment of HER2^+ve^ breast cancer. Encouraging pre-clinical and clinical data indicate that TKIs, alone or in combination, may be particularly useful for the treatment of CNS metastases [11], but further investigation is required to identify the best clinical setting for this class of inhibitors. This has been difficult, however, in the absence of robust models of HER2^+ve^ breast cancer that aggressively spread from the mammary gland to the brain. The TBCP-1 model characterised herein is unique in many respects and fills an important gap in pre-clinical cancer research. We show that TBCP-1 cells express high levels of HER2 but lack expression of hormone receptors. This phenotype is functionally relevant since TBCP-1 cell proliferation is inhibited by several TKIs targeting HER2 but not by anti-oestrogens (Fig. 1). Its clinical relevance is further illustrated by the fact that TBCP-1 tumours and metastases maintain expression of HER2 and response to neratinib in vivo (evidenced by inhibition of HER2 signalling, tumour growth and metastasis) (Figs. 5 and 6) and metastasise to multiple organs commonly colonised by human HER2^+ve^ breast cancer, including the brain (Fig. 2). To our knowledge, TBCP-1 is the only HER2^+ve^ breast cancer model that spreads spontaneously and avidly from the mammary fat pad to brain in immune-competent mice.

We have yet to determine whether high membrane expression of HER2 protein detected by flow cytometry, immunoblotting and immunohistochemistry occurs through gene amplification or alternative mechanisms. While there is generally good concordance between HER2 protein expression and gene amplification in breast cancer patients, high HER2 protein expression without gene amplification occurs in approximately 10% of cases [60]. Importantly, the same study showed no difference in clinical outcome (overall survival) in patients with intermediate (2+) to high (3+) HER2 protein-expressing tumours with or without gene amplification. These observations are consistent with the aggressive nature of the TBCP-1 model. IHC analyses of HER2 status in tumours and brain metastases revealed spatially heterogeneous levels of membrane HER2 expression but overall score consistent with a HER2^+ve^ phenotype. Intra-tumour spatial heterogeneity has been reported also in amplified and non-amplified HER2-overexpressing human breast tumours and could impact on the efficacy of HER2-targeted therapies [61–63]. Hence, it will be important in future studies to assess whether high HER2 expression and spatial intra-tumour heterogeneity may be explained by the differences in gene amplification or due to the differences in microenvironmental factors within specific regions of tumours and metastases.

Sensitivity to neratinib is closely correlated with the level of HER2 expression in human cell lines [16]. While neratinib inhibited the proliferation of TBCP-1, BT474 and SKBR3 cells, BT474 and SKBR3 cells were more strongly inhibited than TBCP-1 cells (IC_50_ ~ 1 nM, 7 nM and 117 nM, respectively). Other mouse and human TNBC or luminal cell lines tested showed varying degree of sensitivity to neratinib that largely correlated with the level of HER2 or EGFR expression, both targeted by neratinib. BT474 and SKBR3 cells express particularly high levels of HER2 due to gene amplification which might explain their greater sensitivity. Alternatively, differences in downstream signalling might contribute in part to the differential response of these cell lines to neratinib. Phosphorylation of receptor tyrosine kinases triggers the activation of multiple signalling pathways that are inhibited by TKIs [15, 64]. In particular, Canonici et al. [16] reported that the sensitivity of HER2-amplified cell lines to neratinib correlates with the extent of p-AKT and p-ERK inhibition. Consistent with this, neratinib almost completely blocked phosphorylation of HER2 as well as downstream activation of AKT and MAPK (ERK-1/2) in SKBR3 cells. In contrast, neratinib significantly inhibited HER2 and AKT phosphorylation but failed to inhibit ERK phosphorylation in TBCP-1 cells (Fig. 3). Interestingly, aberrant activation of downstream ERK signalling through a FOXO-dependent but Ras-independent feedback mechanism has been associated with resistance to HER2-targeting inhibitors [65]. Further work will be required to clarify whether the same feedback loop may operate in TBCP-1 cells and contribute in part to their lower sensitivity to neratinib compared to SKBR3 cells. Our finding from the KEGG pathway analysis showing enrichment of upregulated genes associated with both MAPK and FOXO signalling pathways in neratinib-treated TBCP-1 cells (Additional file 5: Table S2) would be consistent with this possibility.

While neratinib can inhibit HER1, HER2 and HER4, TBCP-1 lack the expression of HER4, and accordingly, neratinib potently inhibited HER2 and partially reduced EGFR phosphorylation (Fig. 3). Moreover, the specific EGFR/HER1 inhibitor, erlotinib, only inhibited SKBR3 cells at high nanomolar concentrations and did not inhibit TBCP-1 proliferation even at micromolar concentrations suggesting that TBCP-1 cell proliferation is not dependent on signalling from EGFR homodimers. Similarly, afatinib, an irreversible inhibitor with ~ 25-fold higher potency against HER1 compared to HER2 [66] only inhibited TBCP-1 at high nanomolar to low micromolar concentrations. Conversely, the selective HER2-reversible inhibitor, tucatinib (> 500-fold selectivity for HER2 compared to HER1) [67, 68], was the second most potent inhibitor (IC_50_, 191 nM) after neratinib (IC_50_, 117 nM). Collectively, these observations indicate that the proliferation of TBCP-1 is critically dependent on HER2 activity and that this receptor is the primary target for neratinib in these cells.

Another key finding from our study is the potent induction of caspase-independent ferroptotic cell death induced by neratinib that could be rescued by the ferroptosis inhibitor liproxstatin-1 (Fig. 4). This response was also evidenced by an uptake of intracellular iron and altered expression of iron metabolism regulators. Amongst all TKIs tested, this property was unique to neratinib and correlated with its superior activity against mouse and human HER2^+ve^ cells. ACSL4, a regulator of fatty acid oxidation or lipid biosynthesis, was identified recently as a biomarker of sensitivity to ferroptosis preferentially expressed in basal-like breast cancer cell lines but also found to be elevated in HER2^+ve^ SKBR3 cells [55]. These observations raise the interesting possibility that the promotion of ferroptosis could be an effective strategy to enhance the efficacy of TKIs against metastatic HER2^+ve^ as well as basal-like breast cancers. In addition, the work of Doll et al. [55] and our study suggest that high levels of ACSL4 or susceptibility to ferroptosis could be predictive biomarkers of neratinib response and facilitate the patient selection. This is currently being investigated.

We were particularly interested in clarifying the properties that are required for TKI targeting EGFR family of receptors to induce ferroptosis. Prior to our study, only sorafenib, a multi-kinase inhibitor that does not target EGFR family members, had been reported to induce ferroptosis in various cancer cell lines although breast cancer lines were not investigated [50]. Lapatinib was shown also to enhance the pro-ferroptotic activity of siramesine, a lysosome destabilising agent, but in agreement with our study, lapatinib alone was not sufficient to induce this response [69]. Comparing the effect of neratinib on multiple mouse and human lines or to other TKIs selected for our study and results reported by others [50, 69] allows us to draw some conclusions. Ferroptosis appears to be correlated with the level of HER2 expression and independent of ERK activation since neratinib induced this response equally well in SKBR3 and TBCP-1 cells but without significant inhibition of ERK-1/2 phosphorylation in TBCP-1 cells (Fig. 3). This conclusion was reached also for sorafenib-induced ferroptosis [50]. None of the reversible inhibitors used herein (erlotinib, lapatinib and tucatinib) induced ferroptosis. Surprisingly, the irreversible pan-inhibitor afatinib alone was unable to illicit this response. It should be noted, however, that afatinib is significantly more potent against HER1 than HER2 [66] and was a poor inhibitor of TBCP-1 cells (IC_50_, 747 nM). On the basis of these observations, we propose that potent and sustained inhibition of HER2 is necessary to induce ferroptosis. Alternatively, targeting multiple downstream signalling pathways may partially circumvent the need for irreversible inhibition of HER2, as suggested by the induction of ferroptosis by the reversible multi-kinase inhibitor sorafenib and enhanced ferroptosis induced by the siramesine + lapatinib combination [50, 69]. Defining the precise mechanisms by which neratinib induces ferroptosis will require further investigation.

Whether the limited efficacy of HER2 inhibitors against brain metastases is due to the poor permeability of inhibitors across the BBB or acquired resistance induced by the brain environment or both is still debated [70]. Pre-clinical studies showing that neratinib inhibits drug efflux pumps have suggested that neratinib may have better retention in the brain [21, 71]. However, in advanced patients with brain involvement, neratinib monotherapy showed only a modest CNS objective response rate (8%), indicating that this may not be the best clinical setting for neratinib [23]. On the other hand, the NEfERT-T trial (NCT00915018) that compared the efficacy of neratinib + paclitaxel versus trastuzumab + paclitaxel as the first-line therapy in women with previously untreated recurrent or metastatic HER2^+ve^ breast cancer showed lower incidence of CNS recurrence in the neratinib-treated compared to the trastuzumab-treated group (8.3% versus 17.3%, respectively) and delayed time to CNS metastases [24] indicating that patients at risk of brain metastasis may benefit from earlier intervention.

The spontaneous nature of the TBCP-1 model of HER2 breast cancer metastasis allowed us to test the efficacy of neratinib in a preventive neoadjuvant and late metastatic setting (Fig. 5). Results from experimental metastasis assay showed a modest improvement in survival and a trend towards the reduced incidence of brain lesions (60% versus 78% in the control group), a result roughly in line with that reported in patients [23]. In contrast, in the neoadjuvant setting, neratinib significantly extended survival and dramatically reduced the overall metastatic burden. Remarkably, none of the mice treated with neratinib had detectable brain lesions compared to 62.5% in the control group. To our knowledge, this is the first pre-clinical demonstration that first-line neratinib neoadjuvant therapy provides significant benefit with regard to CNS recurrence. In our study, metastatic burden after neratinib treatment was analysed at the endpoint. Whether neoadjuvant neratinib reduces brain metastasis by preventing dissemination from the primary tumour, by targeting circulating tumour cells before they home to the brain or by preventing the outgrowth of perivascular micro-metastases in the brain will require more in-depth kinetic studies, including early adjuvant therapy initiated after primary tumour resection. While it is too early to know its impact on brain recurrence in HER2^+ve^ breast cancer patients, the I-SPY2 trial (NCT01042379) evaluating neratinib’s efficacy in the neoadjuvant setting has reported encouraging observations, with an estimated rate of pathological complete response superior to that of the trastuzumab-containing control arm (56% versus 33%, respectively) [72]. Collectively, our results strongly argue for the use of neoadjuvant neratinib and support ongoing trials in this setting.

## Conclusions

We have developed the only mouse model of spontaneous breast cancer metastasis that closely mimics the aggressive spread of HER2^+ve^ breast cancer to the brain and other organs in immune-competent mice. The TBCP-1 model will provide a unique platform to explore new therapies against brain-metastatic HER2^+ve^ breast cancer, including immunotherapies, an area currently understudied. The results from transcriptomic analyses and in vitro investigation identified a new mechanism of action for neratinib and demonstrated that its superior efficacy correlates with the unique ability to induce ferroptosis.

We sought to evaluate the best clinical setting for neratinib in vivo, in particular for the prevention or treatment of brain metastases. Our data show that neratinib neoadjuvant therapy effectively reduces TBCP-1 metastatic burden and extends survival. We propose that prevention of metastatic progression using a neoadjuvant treatment protocol could be more efficacious and provide a greater survival benefit to HER2^+ve^ breast cancer patients than late intervention, particularly against the development of difficult to treat brain metastases.

Despite the potent efficacy of neoadjuvant neratinib, detection of small lung or liver nodules in some mice indicates that resistance can develop in vivo and that combination therapy may be required to completely prevent metastatic progression. Indeed, evidence that resistance can develop after prolonged exposure to neratinib is now emerging, and various mechanisms have been proposed including altered expression of EGFR family members and increased neratinib metabolism, which could reduce its bioavailability [73, 74]. Investigating the mechanisms of resistance to neratinib-induced ferroptosis or the efficacy of combination therapies, including immunotherapy likely to enhance neratinib’s activity [75], will require the use of an appropriate preclinical model, such as TBCP-1, in immune-competent mice.

## Additional files

Additional file 1: **Figure S1.** Schematic of TBCP-1 model development and metastasis assays. (A) Parental TBCP-1 cells were derived from long-term culture of a spontaneously arising mammary tumour from a BALB/C mouse (SMT1). Clonal lines isolated by FACS were selected based on spontaneous metastatic abilities in vivo and phenotype analysis to generate the brain metastatic TBCP-1 line. The morphology of parental and clonal TBCP-1 cells in standard culture is shown in the bottom left panels. Scale bar = 50 μm. Expression of EGFR/HER1, HER2, HER3 and HER4 in TBCP-1 and SKBR3 cells determined by western blotting is shown in bottom middle panels. HER2 membrane expression in TBCP-1 cells was detected by standard flow cytometry (right panel). (B) Experimental metastasis assay. (C) Spontaneous metastasis assay. (TIF 16222 kb)

Additional file 2: **Figure S2.** Principal component analysis of neratinib-treated versus untreated TBCP-1 cells and ferroptotic/apoptotic response to inhibitors. (A) Sub-confluent cultures of TBCP-1 cells were treated for 24 h with vehicle (DMSO) or neratinib (300 nM). Cell viability under those conditions was analysed by flow cytometry. Gating for all events (P1), single cells (P2) and viability (P3) is shown in the top panels and overall viability in control and neratinib-treated cultures, and changes in cell morphology (rounding) induced by neratinib are shown in the bottom panels. (B) Principal component analysis of neratinib-treated versus untreated TBCP-1 cells. Control and neratinib-treated cell lysates were subjected to RNA isolation and sequencing as described in the “Methods” section. (C) Representative images of TBCP-1 cell death induced by neratinib or BH3 mimetics and rescue by ferroptosis or apoptosis inhibitors. Arrows show extensive blebbing induced by BH3 mimetics. Scale bar = 50 μm. (TIF 22771 kb)

Additional file 3: **Figure S3.** Determination of neratinib IC_50_ and pro-ferroptotic activity in mouse and human breast cancer lines and schematic of neratinib treatment protocols. (A) Sensitivity of mouse (left panel) and human (middle panel) breast cancer cell lines to neratinib, and IC_50_ values were determined in short-term (72 h) assays as described in the “Methods” section. Expression of EGFR and HER2 in human lines (right panel) was examined by standard western blotting. The bottom panels show response to neratinib or RSL3 (0.5 μM) treatment in the presence or absence of liproxstatin-1 (2 μM) in the indicated lines. Neratinib was used at 800 nM (67NR), 2.5 μM (4T1.2), 5 μM (MCF-7), 2 nM (BT474) and 500 nM (MDA-MB-231HM). Data show mean ± SD three independent experiment (*n* = 3) done in triplicate wells. **p < 0.01, ****p < 0.001; ns, not significant. (B) Metastatic setting. TBCP-1 cells (5 × 10^5^/100 μl saline) were inoculated into the left cardiac ventricle. Daily treatment by oral gavage commenced 2 days post-inoculation and continued for up to 3 weeks. Mice were sacrificed individually when showing signs of advanced metastatic disease. (C) Neoadjuvant setting. TBCP-1 cells (1 × 10^6^/20 μl) were inoculated orthotopically and daily treatment by oral gavage commenced when tumours reached 100 mm^3^ (~ 1 week). Treatment continued for up to 3 weeks or until tumours were resected when they reached 0.4–0.5 cm^3^ (~ 3 weeks). Mice were sacrificed individually when showing signs of advanced metastatic disease. (TIF 13948 kb)

Additional file 4: **Table S1.** Top 30 enriched “Biological Processes” upregulated genes in neratinib-treated TBCP-1 cells. (DOCX 16 kb)

Additional file 5: **Table S2.** Top 30 enriched KEGG pathways in genes upregulated in neratinib-treated TBCP-1 cells. (DOCX 17 kb)

Additional file 6: **Table S3.** Ferroptosis-associated upregulated genes in neratinib-treated TBCP-1 cells. (DOCX 16 kb)

## Data Availability

The datasets used and/or analysed during the current study are available from the corresponding author on reasonable request.

## Abbreviations

ACSL4: Acyl-CoA synthetase long-chain family member 4
BBB: Blood-brain barrier
CNS: Central nervous system
DMSO: Dimethylsulfoxide
EGFR: Epidermal growth factor receptor
ERα: Oestrogen receptor alpha
FACS: Fluorescence-activated cell sorting
HER2: Human epidermal growth factor receptor 2
IC_50_: Half maximal inhibitory concentration
PR: Progesterone receptor
qPCR: Real-time quantitative PCR
ROS: Reactive oxygen species
RSL3: RAS synthetic lethal 3
SMT1 (cells): Spontaneous mammary tumour 1
SRB: Sulforhodamine B
TBCP-1 (cells): Translational breast cancer program-1
TKI: Tyrosine kinase inhibitors
TNBC: Triple-negative breast cancer
