# Supplementary figures and images for: Neoadjuvant neratinib promotes ferroptosis and inhibits brain metastasis in a novel syngeneic model of spontaneous HER2+ve breast cancer metastasis
Source: Breast Cancer Res. 2019 Aug 13;21:94. doi: 10.1186/s13058-019-1177-1 (PMC6693253; doi:10.1186/s13058-019-1177-1)

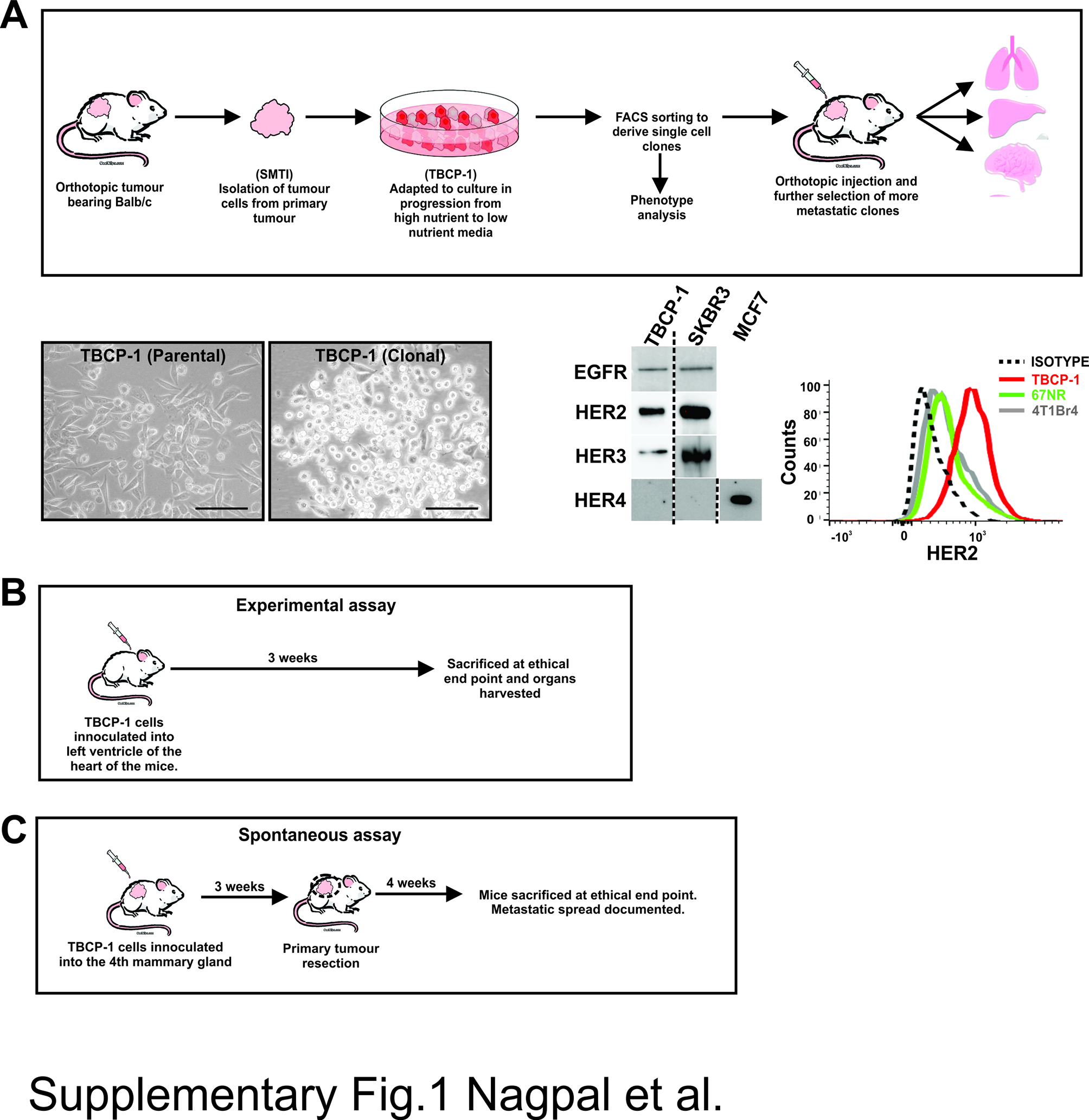

Supplement: Supplementary file 1 — Figure S1. Schematic of TBCP-1 model development and metastasis assays. (A) Parental TBCP-1 cells were derived from long-term culture of a spontaneously arising mammary tumour from a BALB/C mouse (SMT1). Clonal lines isolated by FACS were selected based on spontaneous metastatic abilities in vivo and phenotype analysis to generate the brain metastatic TBCP-1 line. The morphology of parental and clonal TBCP-1 cells in standard culture is shown in the bottom left panels. Scale bar = 50 μm. Expression of EGFR/HER1, HER2, HER3 and HER4 in TBCP-1 and SKBR3 cells determined by western blotting is shown in bottom middle panels. HER2 membrane expression in TBCP-1 cells was detected by standard flow cytometry (right panel). (B) Experimental metastasis assay. (C) Spontaneous metastasis assay. (TIF 16222 kb) [file 13058_2019_1177_MOESM1_ESM.tif]

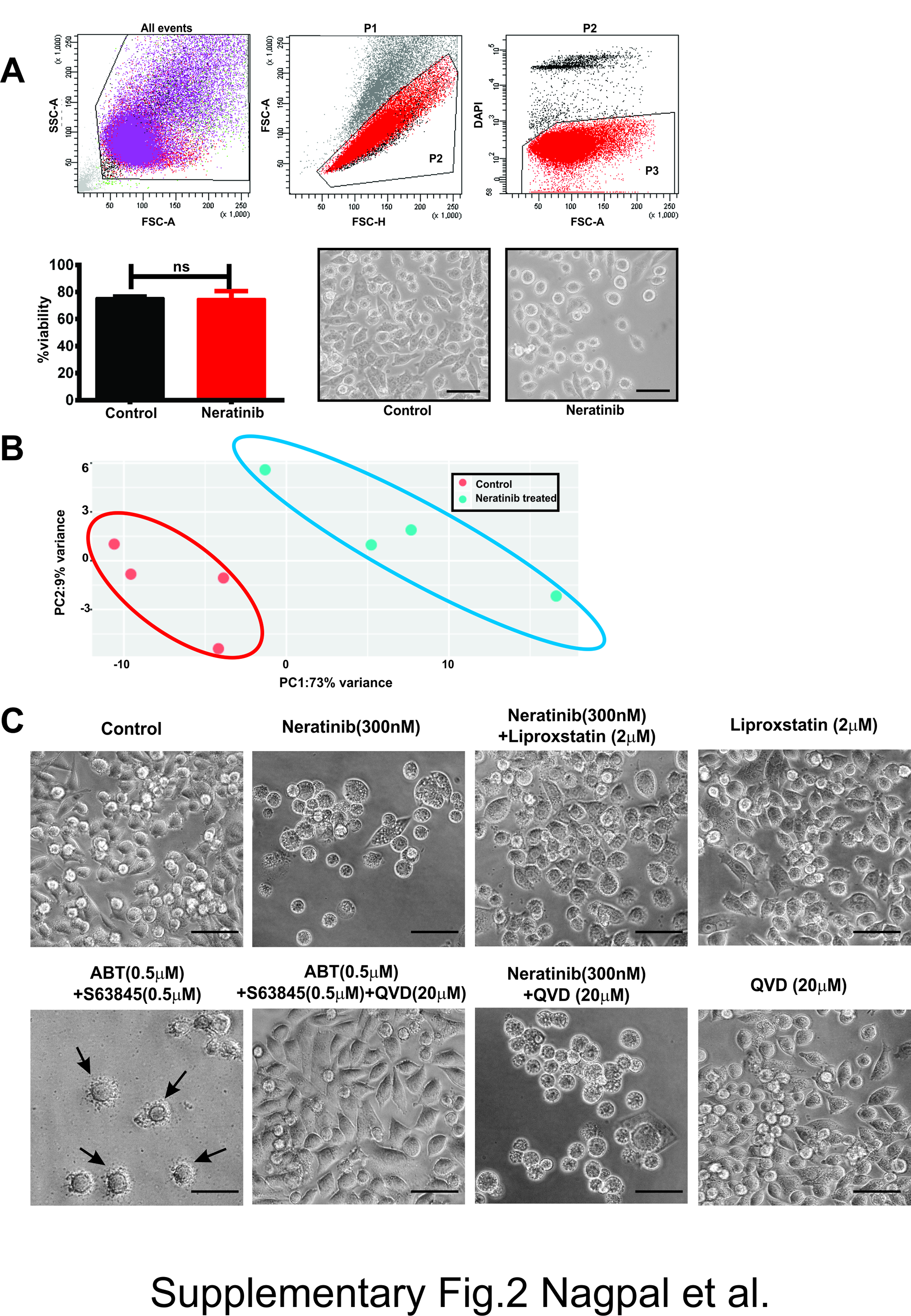

Supplement: Supplementary file 2 — Figure S2. Principal component analysis of neratinib-treated versus untreated TBCP-1 cells and ferroptotic/apoptotic response to inhibitors. (A) Sub-confluent cultures of TBCP-1 cells were treated for 24 h with vehicle (DMSO) or neratinib (300 nM). Cell viability under those conditions was analysed by flow cytometry. Gating for all events (P1), single cells (P2) and viability (P3) is shown in the top panels and overall viability in control and neratinib-treated cultures, and changes in cell morphology (rounding) induced by neratinib are shown in the bottom panels. (B) Principal component analysis of neratinib-treated versus untreated TBCP-1 cells. Control and neratinib-treated cell lysates were subjected to RNA isolation and sequencing as described in the “Methods” section. (C) Representative images of TBCP-1 cell death induced by neratinib or BH3 mimetics and rescue by ferroptosis or apoptosis inhibitors. Arrows show extensive blebbing induced by BH3 mimetics. Scale bar = 50 μm. (TIF 22771 kb) [file 13058_2019_1177_MOESM2_ESM.tif]

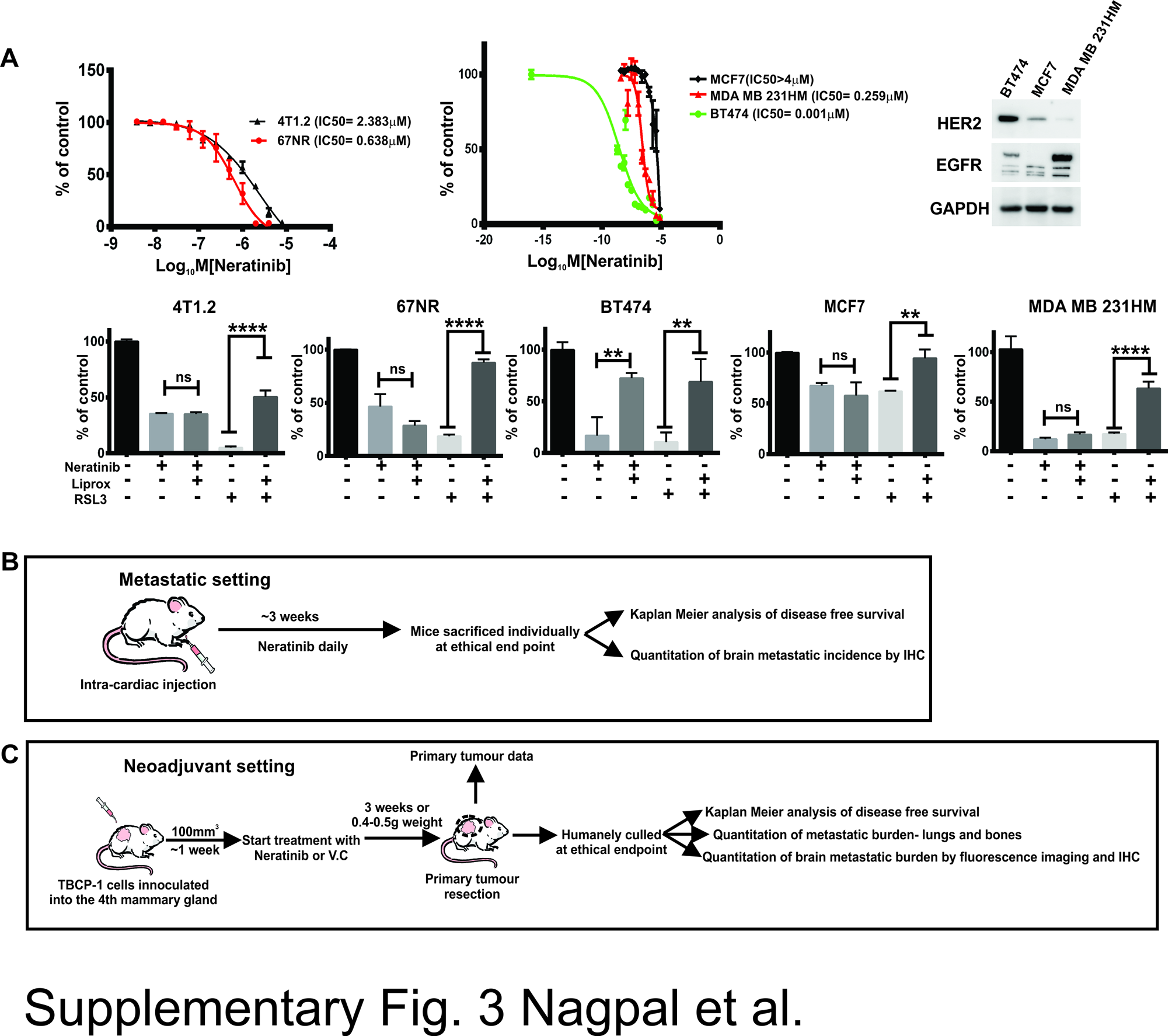

Supplement: Supplementary file 3 — Figure S3. Determination of neratinib IC50 and pro-ferroptotic activity in mouse and human breast cancer lines and schematic of neratinib treatment protocols. (A) Sensitivity of mouse (left panel) and human (middle panel) breast cancer cell lines to neratinib, and IC50 values were determined in short-term (72 h) assays as described in the “Methods” section. Expression of EGFR and HER2 in human lines (right panel) was examined by standard western blotting. The bottom panels show response to neratinib or RSL3 (0.5 μM) treatment in the presence or absence of liproxstatin-1 (2 μM) in the indicated lines. Neratinib was used at 800 nM (67NR), 2.5 μM (4T1.2), 5 μM (MCF-7), 2 nM (BT474) and 500 nM (MDA-MB-231HM). Data show mean ± SD three independent experiment (n = 3) done in triplicate wells. **p < 0.01, ****p < 0.001; ns, not significant. (B) Metastatic setting. TBCP-1 cells (5 × 105/100 μl saline) were inoculated into the left cardiac ventricle. Daily treatment by oral gavage commenced 2 days post-inoculation and continued for up to 3 weeks. Mice were sacrificed individually when showing signs of advanced metastatic disease. (C) Neoadjuvant setting. TBCP-1 cells (1 × 106/20 μl) were inoculated orthotopically and daily treatment by oral gavage commenced when tumours reached 100 mm3 (~ 1 week). Treatment continued for up to 3 weeks or until tumours were resected when they reached 0.4–0.5 cm3 (~ 3 weeks). Mice were sacrificed individually when showing signs of advanced metastatic disease. (TIF 13948 kb) [file 13058_2019_1177_MOESM3_ESM.tif]
