# Supplementary material for: Neoadjuvant neratinib promotes ferroptosis and inhibits brain metastasis in a novel syngeneic model of spontaneous HER2+ve breast cancer metastasis
Source: Breast Cancer Res. 2019 Aug 13;21:94. doi: 10.1186/s13058-019-1177-1 (PMC6693253; doi:10.1186/s13058-019-1177-1)
Supplement: Supplementary file 4 — Table S1. Top 30 enriched “Biological Processes” upregulated genes in neratinib-treated TBCP-1 cells. (DOCX 16 kb) [file 13058_2019_1177_MOESM4_ESM.docx]

**Table S1. Top 20 enriched “Biological Processes” upregulated genes in neratinib-treated TBCP-1 cells.**

| **GO_ID** | **Term** | **N** | **DE** | **P.DE** |
| --- | --- | --- | --- | --- |
| GO:0010468 | regulation of gene expression | 3292 | 340 | 3.30E-08 |
| GO:0051252 | regulation of RNA metabolic process | 2740 | 289 | 7.76E-08 |
| GO:0060255 | regulation of macromolecule metabolic process | 4471 | 439 | 8.34E-08 |
| GO:0019222 | regulation of metabolic process | 4836 | 468 | 1.53E-07 |
| GO:1903506 | regulation of nucleic acid-templated transcription | 2622 | 276 | 2.04E-07 |
| GO:0080090 | regulation of primary metabolic process | 4412 | 431 | 2.27E-07 |
| GO:2001141 | regulation of RNA biosynthetic process | 2630 | 276 | 2.69E-07 |
| GO:0006355 | regulation of transcription, DNA-templated | 2619 | 275 | 2.71E-07 |
| GO:0010604 | positive regulation of macromolecule metabolic process | 2361 | 251 | 3.86E-07 |
| GO:1903508 | positive regulation of nucleic acid-templated transcription | 1145 | 138 | 4.00E-07 |
| GO:0045893 | positive regulation of transcription, DNA-templated | 1145 | 138 | 4.00E-07 |
| GO:1902680 | positive regulation of RNA biosynthetic process | 1151 | 138 | 5.39E-07 |
| GO:0019219 | regulation of nucleobase-containing compound metabolic process | 3037 | 310 | 5.52E-07 |
| GO:0097659 | nucleic acid-templated transcription | 2697 | 280 | 5.61E-07 |
| GO:0031323 | regulation of cellular metabolic process | 4518 | 437 | 5.92E-07 |
| GO:0031326 | regulation of cellular biosynthetic process | 3166 | 321 | 6.09E-07 |
| GO:0006351 | transcription, DNA-templated | 2694 | 279 | 7.36E-07 |
| GO:0009893 | positive regulation of metabolic process | 2565 | 267 | 9.05E-07 |
| GO:0032774 | RNA biosynthetic process | 2712 | 280 | 9.14E-07 |
| GO:0051254 | positive regulation of RNA metabolic process | 1205 | 142 | 9.83E-07 |
