# Supplementary material for: Neoadjuvant neratinib promotes ferroptosis and inhibits brain metastasis in a novel syngeneic model of spontaneous HER2+ve breast cancer metastasis
Source: Breast Cancer Res. 2019 Aug 13;21:94. doi: 10.1186/s13058-019-1177-1 (PMC6693253; doi:10.1186/s13058-019-1177-1)
Supplement: Supplementary file 5 — Table S2. Top 30 enriched KEGG pathways in genes upregulated in neratinib-treated TBCP-1 cells. (DOCX 17 kb) [file 13058_2019_1177_MOESM5_ESM.docx]

**Table S2. Top 20 enriched KEGG pathways in genes up-regulated in neratinib-treated TBCP-1 cells.**

| **Pathway_ID** | **Pathway** | **N** | **DE** | **P.DE** |
| --- | --- | --- | --- | --- |
| path:mmu05215 | Prostate cancer | 91 | 16 | 0.001256372 |
| path:mmu04010 | MAPK signaling pathway | 260 | 31 | 0.007958893 |
| path:mmu04216 | Ferroptosis | 39 | 8 | 0.008090283 |
| path:mmu05225 | Hepatocellular carcinoma | 158 | 21 | 0.008382806 |
| path:mmu04922 | Glucagon signaling pathway | 83 | 13 | 0.009547944 |
| path:mmu05220 | Chronic myeloid leukemia | 76 | 12 | 0.011769239 |
| path:mmu04068 | FoxO signaling pathway | 117 | 16 | 0.015529344 |
| path:mmu04151 | PI3K-Akt signaling pathway | 295 | 33 | 0.015726368 |
| path:mmu05161 | Hepatitis B | 130 | 17 | 0.019422346 |
| path:mmu04115 | p53 signaling pathway | 63 | 10 | 0.019879695 |
| path:mmu04710 | Circadian rhythm | 29 | 6 | 0.020094885 |
| path:mmu05206 | MicroRNAs in cancer | 162 | 20 | 0.021242406 |
| path:mmu05212 | Pancreatic cancer | 74 | 11 | 0.023628357 |
| path:mmu04931 | Insulin resistance | 94 | 13 | 0.025385187 |
| path:mmu01524 | Platinum drug resistance | 75 | 11 | 0.025864225 |
| path:mmu05213 | Endometrial cancer | 57 | 9 | 0.027317431 |
| path:mmu01521 | EGFR tyrosine kinase inhibitor resistance | 76 | 11 | 0.02824953 |
| path:mmu05231 | Choline metabolism in cancer | 96 | 13 | 0.029635403 |
| path:mmu05222 | Small cell lung cancer | 87 | 12 | 0.031584962 |
| path:mmu04142 | Lysosome | 117 | 15 | 0.031741052 |
