# Supplementary material for: Neoadjuvant neratinib promotes ferroptosis and inhibits brain metastasis in a novel syngeneic model of spontaneous HER2+ve breast cancer metastasis
Source: Breast Cancer Res. 2019 Aug 13;21:94. doi: 10.1186/s13058-019-1177-1 (PMC6693253; doi:10.1186/s13058-019-1177-1)
Supplement: Supplementary file 6 — Table S3. Ferroptosis-associated upregulated genes in neratinib-treated TBCP-1 cells. (DOCX 16 kb) [file 13058_2019_1177_MOESM6_ESM.docx]

**Table S3. Ferroptosis-associated upregulated genes in neratinib treated TBCP-1 cells.**

| **mEntrez ID** | **MGI symbol** | **hEntrezGene ID** | **Description** |
| --- | --- | --- | --- |
| 53945 | Slc40a1 | 30061 | solute carrier family 40 (iron-regulated transporter), member 1 |
| 12870 | Cp | 1356 | ceruloplasmin |
| 26570 | Slc7a11 | 23657 | solute carrier family 7 (cationic amino acid transporter, y+ system), member 11 |
| 216739 | Acsl6 | 23305 | acyl-CoA synthetase long-chain family member 6 |
| 18521 | Pcbp2 | 5094 | poly(rC) binding protein 2 |
| 14319 | Fth1 | 2495 | ferritin heavy chain 1 |
| 50790 | Acsl4 | 2182 | acyl-CoA synthetase long-chain family member 4 |
| 20229 | Sat1 | 6303 | spermidine/spermine N1-acetyl transferase 1 |
